# Supplementary material for: Transcriptomics Reveals Host-Dependent Differences of Polysaccharides Biosynthesis in Cynomorium songaricum
Source: Molecules. 2021 Dec 22;27(1):44. doi: 10.3390/molecules27010044 (PMC8746405; doi:10.3390/molecules27010044)
Supplement: Supplementary file 1 [file molecules-27-00044-s001.zip › molecules-1465708-supplementary.pdf]

Supplementary Materials

Table S1. Summary of sequencing data for *Cynomorium songaricum* transcriptome.

	CR	CS
**Unfiltered data**		
Data of reads number (million)	51.31	46.89
Reads length	150	150
GC (%)	47.61	47.78
Data of reads number×read length (million)	7,696	7,033
Q20 (%)	96.77	96.67
Q30 (%)	91.47	91.29
**Filtered data**[1]		
Data of reads number (million)	51.24	46.80
Data of reads number×read length (million)	7,686	7,020
Q20 (%)	96.82	96.72
Q30 (%)	91.54	91.36
**Mapped data**[2]		
Data of unique mapped reads (million)	42.46	39.45
Data of multiple mapped reads (million)	1.61	1.39
Mapping ratio (%)	89.90	90.31
**Compiled data**		
Total number of unigenes	95,126	
Total Length (bp) (million)	68.96	
N50 (bp)	1,086	
Max length (bp)	15,090	
Min length (bp)	201	
Average Length (bp)	724	
GC content (%)	42.03	

[1] Reads with a quality score < 30 and length < 60 bp were excluded;

[2] Mapping ratio = (Unique mapped reads + Multiple mapped reads) / Filtered reads.

Table S2. Primary metabolism genes differentially expressed in CR and CS.

Gene name	Protein name	log_2 Ratio (CR/CS)
**Carbohydrate metabolism**		
N/A	Glycosyl hydrolase 5 family protein	2.66
A6	Probable glucan endo-1,3-beta-glucosidase A6	1.51
AGAL2	Alpha-galactosidase 2	-1.96
Aldoa	Fructose-bisphosphate aldolase A	-10.68
ALG10	Dol-P-Glc:Glc(2)Man(9)GlcNAc(2)-PP-Dol alpha-1,2-glucosyltransferase	-1.23
At1g27200	Glycosyltransferase family 92 protein At1g27200	-5.03
At1g32860	Glucan endo-1,3-beta-glucosidase 11	3.20
At1g48100	Polygalacturonase At1g48100	2.40
At1g64760	Glucan endo-1,3-beta-glucosidase 8	1.83
At2g43610	Endochitinase At2g43610	2.72
At2g43620	Endochitinase At2g43620	3.38
At2g43860	Polygalacturonase	-2.32
At2g01630	Glucan endo-1,3-beta-glucosidase 3	2.19
At2g27500	Glucan endo-1,3-beta-glucosidase 14	1.20
At2g31390	Probable fructokinase-1	2.94
At3g08590	Probable 2,3-bisphosphoglycerate-independent phosphoglycerate mutase 2	2.42
At3g05260	Glucose and ribitol dehydrogenase homolog 2	-1.04
At4g26390	Probable pyruvate kinase, cytosolic isozyme	3.58
At4g29360	Glucan endo-1,3-beta-glucosidase 12	1.63
AXY4	ALTERED XYLOGLUCAN 4	2.26
AXY4L	Protein ALTERED XYLOGLUCAN 4-like	-1.17
BACOVA_02659	Beta-glucosidase BoGH3B	1.61
BG	Basic 7S globulin	3.26
BGAL12	Beta-galactosidase	-1.00
BGAL5	Beta-galactosidase 5	-1.32
BGAL7	Beta-galactosidase 7	-3.29
BGLU23	Beta-glucosidase 23	-2.57
BGLU25	Probable inactive beta-glucosidase 25	3.12
BGLU44	Beta-glucosidase 44	-1.08
BGLU6	Beta-glucosidase 6	-1.53
BXL4	Beta-D-xylosidase 4	1.78
BXL5	Probable beta-D-xylosidase 5	-2.84
CAISE5	Glucose and ribitol dehydrogenase	5.35
CALS10	Callose synthase 10	3.86
CALS9	Callose synthase 9	8.81
Cbr2	Carbonyl reductase [NADPH] 2	-8.62
CEMIP	Cell migration-inducing and hyaluronan-binding protein	-10.45
Cemip	Cell migration-inducing and hyaluronan-binding protein	-10.21
ChiC	Class V chitinase	-1.26
CHIT3	Hevamine-A	3.75
CWINV1	Beta-fructofuranosidase, insoluble isoenzyme CWINV1	1.40
CYFBP	Fructose-1,6-bisphosphatase, cytosolic	2.29
CYT1	Mannose-1-phosphate guanylyltransferase 1	3.29
DSP4	Phosphoglucan phosphatase DSP4, amyloplastic	-1.95

ENO	Enolase	2.10
ENO1	Alpha-enolase	-8.14
ENO1	Alpha-enolase	-9.56
eno1	Alpha-enolase	9.54
ENO2	Bifunctional enolase 2/transcriptional activator	3.04
EP3	Endochitinase EP3	-4.70
FBA3	Fructose-bisphosphate aldolase 3, chloroplastic	1.73
FBA5	Fructose-bisphosphate aldolase 5, cytosolic	4.09
FBA6	Fructose-bisphosphate aldolase 6, cytosolic	4.07
FBA8	Fructose-bisphosphate aldolase 8, cytosolic	3.08
GAE1	UDP-glucuronate 4-epimerase 1	4.21
GAE6	UDP-glucuronate 4-epimerase 6	4.73
GALM	Aldose 1-epimerase	1.34
GapA	Glyceraldehyde-3-phosphate dehydrogenase A, chloroplastic	8.83
GAPA1	Glyceraldehyde-3-phosphate dehydrogenase GAPA1, chloroplastic	5.47
GAPA2	Glyceraldehyde-3-phosphate dehydrogenase GAPA2, chloroplastic	4.37
GAPB	Glyceraldehyde-3-phosphate dehydrogenase GAPB, chloroplastic	7.25
GAPC	Glyceraldehyde-3-phosphate dehydrogenase, cytosolic	3.25
GAPC1	Glyceraldehyde-3-phosphate dehydrogenase GAPC1, cytosolic	3.21
GAPC2	Glyceraldehyde-3-phosphate dehydrogenase GAPC2, cytosolic	3.42
GAPDH	Glyceraldehyde-3-phosphate dehydrogenase	-10.12
GAPDH	Glyceraldehyde-3-phosphate dehydrogenase	-9.94
gapdh-2	Glyceraldehyde-3-phosphate dehydrogenase 2	10.76
GATL1	Probable galacturonosyltransferase-like 1	1.73
GLC1	Glucan endo-1,3-beta-glucosidase	-3.24
GMD1	GDP-mannose 4,6 dehydratase 1	2.98
GN1	Lichenase	-1.75
GOLS2	Galactinol synthase 2	-1.23
GPD3	Glyceraldehyde-3-phosphate dehydrogenase 3	2.24
GSVIVT 00026920001	Probable polygalacturonase	1.15
Gyg1	Glycogenin-1	-1.24
INVA	Acid beta-fructofuranosidase	2.29
INVF	Probable alkaline/neutral invertase F	1.35
IRX7	Probable glucuronoxylan glucuronosyltransferase IRX7	1.46
IRX15	Protein IRREGULAR XYLEM 15	-1.28
LDHA	L-lactate dehydrogenase B	2.56
LDHA	L-lactate dehydrogenase A	3.14
LDHA	L-lactate dehydrogenase A chain	-8.19
ldhba	L-lactate dehydrogenase B-A chain	9.98
MAN5	Mannan endo-1,4-beta-mannosidase 5	1.72
Mat2a	S-adenosylmethionine synthase isoform type-2	9.45
MIOX1	Inositol oxygenase 1	-2.64
MUR1	GDP-mannose 4,6 dehydratase 2	3.21
MSR2	Protein MANNAN SYNTHESIS-RELATED 2	1.24
NANA	Aspartic proteinase NANA, chloroplast	-3.64
OFUT20	O-fucosyltransferase 20	-2.53
OFUT23	O-fucosyltransferase 23	-1.86
OFUT27	O-fucosyltransferase 27	-1.19
OFUT35	O-fucosyltransferase 35	1.14
OFUT9	O-fucosyltransferase 9	1.16

PF14_0425	Fructose-bisphosphate aldolase	3.54
pfk1	ATP-dependent 6-phosphofructokinase	2.16
PFK3	ATP-dependent 6-phosphofructokinase 3	3.26
PGK1	Phosphoglycerate kinase 1	-8.29
PGAM1	Phosphoglycerate mutase 1	-8.57
PGMP	Phosphoglucomutase, chloroplastic	-1.70
PKM	Pyruvate kinase PKM	-10.01
PMI2	Mannose-6-phosphate isomerase 2	1.61
pyk1	Pyruvate kinase	1.71
RFS2	Probable galactinol--sucrose galactosyltransferase 2	1.76
RFS5	Probable galactinol--sucrose galactosyltransferase 5	-1.56
RFS6	Probable galactinol--sucrose galactosyltransferase 6	2.85
SBE2.2	1,4-alpha-glucan-branching enzyme 2-2, chloroplastic/amyloplastic	-1.79
SETH3	Probable arabinose 5-phosphate isomerase	8.82
sgl	UDP-glucose 6-dehydrogenase	8.35
SS2	Granule-bound starch synthase 2, chloroplastic/amyloplastic	4.06
tal	Transaldolase	2.51
tal	Transaldolase	1.76
TBL33	Protein trichome birefringence-like 33	1.44
Tkfc	Triokinase/FMN cyclase	1.21
TPPF	Probable trehalose-phosphate phosphatase F	2.27
TPPJ	Probable trehalose-phosphate phosphatase J	2.48
TPS11	Probable alpha,alpha-trehalose-phosphate synthase [UDP-forming] 11	3.34
TPS7	Probable alpha,alpha-trehalose-phosphate synthase [UDP-forming] 7	-1.40
TPS9	Probable alpha,alpha-trehalose-phosphate synthase [UDP-forming] 9	8.76
UGP1	UTP--glucose-1-phosphate uridylyltransferase 1	2.80
UGT71A15	UDP-glycosyltransferase 71A15	2.93
UGT73B1	UDP-glycosyltransferase 73B1	3.50
UGT73B5	UDP-glycosyltransferase 73B5	4.69
UGT74C1	UDP-glycosyltransferase 74C1	-3.17
UGT85A24	7-deoxyloganetin glucosyltransferase	-4.34
UGT87A1	UDP-glycosyltransferase 87A1	-1.80
UGT88F5	UDP-glycosyltransferase 88F5	-1.49
UGT89A2	UDP-glycosyltransferase 89A2	-1.40
UGT94E5	Beta-D-glucosyl crocetin beta-1,6-glucosyltransferase	-7.26
USP	UDP-sugar pyrophosphorylase	1.05
UXS3	UDP-glucuronic acid decarboxylase 3	3.65
xlnA	Endo-1,4-beta-xylanase A	1.81
XTH18	Probable xyloglucan endotransglucosylase/hydrolase protein 18	1.88
XTH22	Xyloglucan endotransglucosylase/hydrolase protein 22	2.78
XTH23	Probable xyloglucan endotransglucosylase/hydrolase protein 23	3.22
XTH27	Probable xyloglucan endotransglucosylase/hydrolase protein 27	2.69
XTH30	Probable xyloglucan endotransglucosylase/hydrolase protein 30	-3.79
XTH32	Probable xyloglucan endotransglucosylase/hydrolase protein 32	2.07
XTH9	Xyloglucan endotransglucosylase/hydrolase protein 9	3.04
XXT3	Probable xyloglucan 6-xylosyltransferase 3	2.71
N/A	2,3-bisphosphoglycerate-dependent phosphoglycerate mutase	-1.31
**Lipids metabolism**		
N/A	Lipase	1.01
AAE18	Probable acyl-activating enzyme 18, peroxisomal	-1.33

accD	Acetyl-coenzyme A carboxylase carboxyl transferase subunit beta, chloroplastic	-1.03
accD	Acetyl-coenzyme A carboxylase carboxyl transferase subunit beta, chloroplastic	-1.08
ACL1.1	Acyl carrier protein 1, chloroplastic	-1.57
ACX3	Acyl-coenzyme A oxidase 3, peroxisomal	-2.09
ACX4	Acyl-coenzyme A oxidase 4, peroxisomal	3.23
ACLB-2	ATP-citrate synthase beta chain protein 2	-1.04
APA1	Aspartic proteinase A1	1.72
APG	GDSL esterase/lipase APG	6.15
AT1	Long-chain-alcohol O-fatty-acyltransferase	-2.53
At1g06800	Phospholipase A1-Igamma1, chloroplastic	4.56
At1g11820	Glucan endo-1,3-beta-glucosidase 1	2.09
At1g54790	GDSL esterase/lipase At1g54790	-1.14
At1g71691	GDSL esterase/lipase At1g71691	6.37
At3g26430	GDSL esterase/lipase At3g26430	-9.34
At3g48460	GDSL esterase/lipase At3g48460	-3.07
At4g16820	Phospholipase A1-Ibeta2, chloroplastic	-4.78
At4g18970	GDSL esterase/lipase At4g18970	4.00
At4g26790	GDSL esterase/lipase At4g26790	2.97
At5g03610	GDSL esterase/lipase At5g03610	1.06
At5g33370	GDSL esterase/lipase At5g33370	5.36
At5g41890	GDSL esterase/lipase At5g41890	2.52
At5g42170	GDSL esterase/lipase At5g42170	-1.14
At5g67130	PI-PLC X domain-containing protein At5g67130	-1.82
CLS	Cardiolipin synthase (CMP-forming), mitochondrial	1.79
CPRD49	GDSL esterase/lipase CPRD49	2.23
CUT1	3-ketoacyl-CoA synthase 6	1.44
CYP86A2	Cytochrome P450 86A2	4.23
DCI1	Delta(3,5)-Delta(2,4)-dienoyl-CoA isomerase, peroxisomal	-1.03
DGAT1C	Diacylglycerol O-acyltransferase 1C	-3.71
ECI2	Enoyl-CoA delta isomerase 2, peroxisomal	3.94
FAB2	Stearoyl-[acyl-carrier-protein] 9-desaturase, chloroplastic	3.30
fabG	3-oxoacyl-[acyl-carrier-protein] reductase FabG	4.84
FABP3	Fatty acid-binding protein, heart	9.18
FAD2	Omega-6 fatty acid desaturase, endoplasmic reticulum	2.09
FAD6	Omega-6 fatty acid desaturase, chloroplastic	8.52
FAD6	Omega-6 fatty acid desaturase, chloroplastic	9.09
FAD6	Omega-6 fatty acid desaturase, chloroplastic	9.22
FAD7	sn-2 acyl-lipid omega-3 desaturase (ferredoxin), chloroplastic	-2.82
FAD7A-1	Omega-3 fatty acid desaturase, chloroplastic	-2.03
FAH1	Dihydroceramide fatty acyl 2-hydroxylase FAH1	2.17
FAR2	Fatty acyl-CoA reductase 2	-1.49
FAR3	Fatty acyl-CoA reductase 3	-1.71
FDH	3-ketoacyl-CoA synthase 10	3.11
GDPDL7	Glycerophosphodiester phosphodiesterase GDPDL7	1.32
HACL	2-hydroxyacyl-CoA lyase	4.51
INPS1	Inositol-3-phosphate synthase	1.39
KCS1	3-ketoacyl-CoA synthase 1	3.18
KCS11	3-ketoacyl-CoA synthase 11	-1.50
KCS20	3-ketoacyl-CoA synthase 20	-1.87

LACS2	Long chain acyl-CoA synthetase 2	1.90
LACS4	Long chain acyl-CoA synthetase 4	2.14
LAG2	LAG1 longevity assurance homolog 2	1.13
lip	Lipase	1.67
LIP1	Triacylglycerol lipase 1	1.13
LPAT4	Probable 1-acyl-sn-glycerol-3-phosphate acyltransferase 4	-1.66
LYPLA1	Acyl-protein thioesterase 1	-1.32
MFP2	Glyoxysomal fatty acid beta-oxidation multifunctional protein MFP-a	-1.59
MTACP1	Acyl carrier protein 1, mitochondrial	1.68
MTM1	Phosphatidylinositol-3-phosphatase myotubularin-1	-2.25
NADP-ME2	NADP-dependent malic enzyme	-1.00
NADP-ME4	NADP-dependent malic enzyme 4, chloroplastic	4.61
PAS2A	Very-long-chain (3R)-3-hydroxyacyl-CoA dehydratase PASTICCINO 2A	-1.40
PED1	3-ketoacyl-CoA thiolase 2, peroxisomal	2.62
PLC6	Phosphoinositide phospholipase C 6	1.97
PLDDELTA	Phospholipase D delta	1.47
PLP2	Patatin-like protein 2	3.08
PLP9	Probable inactive patatin-like protein 9	1.33
SDRA	Short-chain dehydrogenase/reductase SDRA	5.15
SEI1	Seipin-1	-1.96
SLD2	Acyl-lipid (9-3)-desaturase	1.33
WRI1	Ethylene-responsive transcription factor WRI1	-3.65
**Amino acids metabolism**		
3-Apr	5'-adenylylsulfate reductase 3, chloroplastic	1.31
ADT3	Arogenate dehydratase 3, chloroplastic	-1.15
AGT1	Serine--glyoxylate aminotransferase	9.13
ALAAT1	Alanine aminotransferase 1, mitochondrial	3.36
ARGAH1	Arginase 1, mitochondrial	8.29
ASK5	Shaggy-related protein kinase epsilon	-1.69
ASN1	Asparagine synthetase [glutamine-hydrolyzing] 1	1.49
ASP1	Aspartate aminotransferase, mitochondrial	-1.03
ASP1	Aspartic proteinase Asp1	1.10
ASP2	Aspartate aminotransferase, cytoplasmic isozyme 1	2.28
ASP3	Aspartate aminotransferase 3, chloroplastic	1.06
ASPG1	Protein ASPARTIC PROTEASE IN GUARD CELL 1	1.27
At4g29120	Probable 3-hydroxyisobutyrate dehydrogenase-like 1, mitochondrial	4.11
BCAT2	Branched-chain-amino-acid aminotransferase 2, chloroplastic	-1.74
BCDH	2-oxoisovalerate dehydrogenase subunit beta 1, mitochondrial	3.71
CM1	Chorismate mutase 1, chloroplastic	-2.25
CM2	Chorismate mutase 2	-1.22
CS1	Chorismate synthase 1, chloroplastic	-1.28
ctps	CTP synthase	3.55
DHAPS-1	Phospho-2-dehydro-3-deoxyheptonate aldolase 2, chloroplastic	-1.05
DHDPS2	4-hydroxy-tetrahydrodipicolinate synthase 2, chloroplastic	3.55
DHS1	Phospho-2-dehydro-3-deoxyheptonate aldolase 1, chloroplastic	2.65
EMB3004	Bifunctional 3-dehydroquinate dehydratase/shikimate dehydrogenase, chloroplastic	5.49
GAD1	Glutamate decarboxylase 1	2.07
GDCST	Aminomethyltransferase, mitochondrial	1.49
GDH2	Glycine cleavage system H protein 2, mitochondrial	1.02

GDH2	Glutamate dehydrogenase 2	2.99
GEK1	D-aminoacyl-tRNA deacylase	4.96
GGAT1	Glutamate--glyoxylate aminotransferase 1	4.16
GGH3	Probable gamma-glutamyl hydrolase 3	-1.02
GGT3	Glutathione hydrolase 3	-3.17
GLN1-2	Glutamine synthetase cytosolic isozyme 1-2	-3.94
GLT1	Glutamate synthase 1 [NADH], chloroplastic	2.70
GS1-1	Glutamine synthetase cytosolic isozyme 1	-1.38
GS1-2	Glutamine synthetase nodule isozyme	2.21
GSH2	Glutathione synthetase, chloroplastic	1.17
GSTU10	Glutathione S-transferase U10	5.55
GSTU19	Glutathione S-transferase U19	2.39
GSTU8	Glutathione S-transferase U8	4.52
HGO	Homogentisate 1,2-dioxygenase	3.15
HISN3	1-(5-phosphoribosyl)-5-[(5-phosphoribosylamino)methylideneamino] imidazole-4-carboxamide isomerase, chloroplastic	-1.33
HISN7	Bifunctional phosphatase IMPL2, chloroplastic	-1.58
HMGCL	Hydroxymethylglutaryl-CoA lyase, mitochondrial	1.37
HMT1	Homocysteine S-methyltransferase 1	4.13
ilv-2	Ketol-acid reductoisomerase, mitochondrial	4.05
IPMS1	2-isopropylmalate synthase 1, chloroplastic	3.11
ISS1	Aromatic aminotransferase ISS1	-1.85
IVD	Isovaleryl-CoA dehydrogenase, mitochondrial	8.93
LCD	L-cysteine desulfhydrase	8.27
LIP1	Lipoyl synthase, mitochondrial	3.48
lys3	Saccharopine dehydrogenase [NAD(+), L-lysine-forming]	4.18
MCCB	Methylcrotonoyl-CoA carboxylase beta chain, mitochondrial	8.93
NAA10	N-terminal acetyltransferase A complex catalytic subunit NAA10	2.13
NMT1	Glycylpeptide N-tetradecanoyltransferase 1	1.41
NMT2	Phosphomethylethanolamine N-methyltransferase	1.74
NSFBx	Probable F-box protein At5g04010	9.23
OXP1	5-oxoprolinase	-1.20
P4H2	Prolyl 4-hydroxylase 2	3.07
P4H3	Probable prolyl 4-hydroxylase 3	3.10
P5CS	Delta-1-pyrroline-5-carboxylate synthase	1.36
P5CSB	Delta-1-pyrroline-5-carboxylate synthase B	3.59
PAL	Phenylalanine ammonia-lyase	-1.19
PCAS-1	Bifunctional L-3-cyanoalanine synthase/cysteine synthase 1, mitochondrial	1.71
PDX13	Pyridoxal 5'-phosphate synthase subunit PDX1.3	4.34
PGDH2	D-3-phosphoglycerate dehydrogenase 2, chloroplastic	2.70
POX2	Proline dehydrogenase 2, mitochondrial	1.40
PYD1	Dihydropyrimidine dehydrogenase (NADP(+)), chloroplastic	1.02
SAMDC4	S-adenosylmethionine decarboxylase proenzyme 4	2.02
serA	D-3-phosphoglycerate dehydrogenase	6.85
SHM1	Serine hydroxymethyltransferase 1, mitochondrial	8.97
SK3	Shikimate kinase 3, chloroplastic	-1.72
TGM2	Protein-glutamine gamma-glutamyltransferase 2	-9.92
THA1	Probable low-specificity L-threonine aldolase 1	4.68
TYRAAT1	Arogenate dehydrogenase 1, chloroplastic	-2.25
TYRDC-2	Tyrosine decarboxylase 2	4.17

Protein metabolism

Acot13	Acyl-coenzyme A thioesterase 13	1.30
AP25	Aspartyl protease 25	1.45
Apoe	Apolipoprotein E	-9.95
At1g17710	Inorganic pyrophosphatase 2	-3.42
At1g55760	BTB/POZ domain-containing protein At1g55760	-1.02
At1g67340	F-box protein At1g67340	1.67
At3g53970	Probable proteasome inhibitor	8.48
At4g03415	Probable protein phosphatase 2C 52	1.05
At4g22670	FAM10 family protein At4g22670	8.44
At5g04500	Glucosamine inositolphosphorylceramide transferase 1	1.95
At5g03795	Probable glycosyltransferase At5g03795	-1.39
At5g17580	BTB/POZ domain-containing protein At5g17580	1.78
At5g48800	BTB/POZ domain-containing protein At5g48800	1.53
ATJ3	Chaperone protein dnaJ 3	2.53
ATL23	E3 ubiquitin-protein ligase ATL23	1.26
ATL4	E3 ubiquitin-protein ligase ATL4	1.72
ATL5	RING-H2 finger protein ATL5	-2.38
ATL54	RING-H2 finger protein ATL54	2.77
ATL64	RING-H2 finger protein ATL64	8.58
ATL65	RING-H2 finger protein ATL65	2.63
ATL72	RING-H2 finger protein ATL72	-1.90
B3GALT12	Probable beta-1,3-galactosyltransferase 12	1.25
B3GALT7	Beta-1,3-galactosyltransferase 7	1.35
BAG5	BAG family molecular chaperone regulator 5, mitochondrial	1.49
BRG3	Probable BOI-related E3 ubiquitin-protein ligase 3	1.85
CCT5	T-complex protein 1 subunit epsilon	3.01
CDC48A	Cell division control protein 48 homolog A	2.83
CDKB1-1	Cyclin-dependent kinase B1-1	1.86
CDKG1	Cyclin-dependent kinase G1	3.40
CDR1	Aspartic proteinase CDR1	1.83
CHIP	E3 ubiquitin-protein ligase CHIP	9.02
CLPB1	Chaperone protein ClpB1	9.68
CLPB4	Chaperone protein ClpB4, mitochondrial	4.54
CLPP5	ATP-dependent Clp protease proteolytic subunit 5, chloroplastic	-2.82
cprG	Cysteine proteinase 7	8.28
CTSC	Dipeptidyl peptidase 1	-8.82
CTSL	Cathepsin L1	8.80
CYP18-1	Peptidyl-prolyl cis-trans isomerase CYP18-1	-2.67
CYPA	Peptidyl-prolyl cis-trans isomerase	10.51
degQ	Periplasmic pH-dependent serine endoprotease DegQ	-1.15
DIS1	E3 ubiquitin-protein ligase DIS1	1.29
DJ1A	Protein DJ-1 homolog A	8.67
dnaJ	Chaperone protein DnaJ	-2.03
DRIP2	E3 ubiquitin protein ligase DRIP2	4.34
EDA2	Probable serine protease EDA2	-1.85
EOGT	EGF domain-specific O-linked N-acetylglucosamine transferase	3.23
EOGT	EGF domain-specific O-linked N-acetylglucosamine transferase	1.01
FKBP65	Peptidyl-prolyl cis-trans isomerase FKBP65	4.88
GALT29A	Beta-1,6-galactosyltransferase GALT29A	-3.71
GT11	Probable xyloglucan galactosyltransferase GT11	1.70

GT17	Probable xyloglucan galactosyltransferase GT17	-1.30
HOP3	Hsp70-Hsp90 organizing protein 3	8.47
HPTG1	Hydroxyproline O-galactosyltransferase HPGT1	-2.33
IGFBP7	Insulin-like growth factor-binding protein 7	-9.46
MMP2	72 kDa type IV collagenase	10.00
NFP	Serine/threonine receptor-like kinase NFP	-2.88
Os03g0144800	Xyloglucan galactosyltransferase KATAMARI1 homolog	1.25
Os04g0670200	Oryzain beta chain	-1.24
PAA2	Proteasome subunit alpha type-6-B	2.61
PDK	[Pyruvate dehydrogenase (acetyl-transferring)] kinase, mitochondrial	1.18
PP2B1	F-box protein PP2-B1	4.84
PPIA	Peptidyl-prolyl cis-trans isomerase A	-10.05
PRMT3	Probable protein arginine N-methyltransferase 3	1.15
PUB25	U-box domain-containing protein 25	3.59
RD19D	Probable cysteine protease RD19D	-1.18
RD21C	Probable cysteine protease RD21C	2.34
RF178	Probable E3 ubiquitin-protein ligase BAH1-like	1.19
RMA1	E3 ubiquitin-protein ligase RMA1	3.59
RMA1H1	E3 ubiquitin-protein ligase RMA1H1	-2.15
RMR2	Receptor homology region, transmembrane domain- and RING domain-containing protein 2	2.62
RPN11	26S proteasome non-ATPase regulatory subunit 14 homolog	2.49
RPN6	26S proteasome non-ATPase regulatory subunit 11 homolog	8.55
SCPL25	Serine carboxypeptidase-like 25	4.76
SCPL29	Serine carboxypeptidase-like 29	-1.49
SCPL31	Serine carboxypeptidase-like 31	-3.64
SCPL40	Serine carboxypeptidase-like 40	-2.82
SCPL45	Serine carboxypeptidase-like 45	1.53
SGR9	E3 ubiquitin-protein ligase SGR9, amyloplastic	3.03
SKIP2	F-box protein SKIP2	3.55
SKIP32	F-box protein 7	-1.07
SMXL3	Protein SMAX1-LIKE 3	1.29
SMXL4	Protein SMAX1-LIKE 4	2.01
TUN	UDP-glycosyltransferase TURAN	-1.54
UBC	Polyubiquitin-C	-3.83
UBC1	Ubiquitin-conjugating enzyme E2 1	2.81
UBC10	Ubiquitin-conjugating enzyme E2 10	2.46
UBC11	Ubiquitin-conjugating enzyme E2 11	3.56
UBC16	Probable ubiquitin-conjugating enzyme E2 16	-1.73
UBC19	Ubiquitin-conjugating enzyme E2 19	3.32
UBC36	Ubiquitin-conjugating enzyme E2 36	-1.38
UBC9	SUMO-conjugating enzyme UBC9	2.05
UBQ10	Polyubiquitin 10	5.39
XB3	E3 ubiquitin-protein ligase XB3	2.73
**Nucleic acid metabolism**		
AAH	Allantoate deiminase	3.44
AAH2	Allantoate deiminase 2	-1.29
CSLA9	Glucomannan 4-beta-mannosyltransferase 9	1.37
CTGF	CCN family member 2	-9.94
DUT	Deoxyuridine 5'-triphosphate nucleotidohydrolase	2.00
TK1A	Thymidine kinase a	1.52

PAB2	Polyadenylate-binding protein 2	2.74
qtrt1	Queuine tRNA-ribosyltransferase catalytic subunit 1	1.79
RNR1	Ribonucleoside-diphosphate reductase large subunit	1.20
RNS2	Ribonuclease 2	2.84
TSO2	Ribonucleoside-diphosphate reductase small chain C	1.79
txn	Thioredoxin	9.32
XRN3	5'-3' exoribonuclease 3	-11.92
**Others**		
ABHD17B	Alpha/beta hydrolase domain-containing protein 17B	1.62
abhd17c	Alpha/beta hydrolase domain-containing protein 17C	3.89
AKR1	Probable aldo-keto reductase 1	-2.76
AKR4C11	Aldose reductase	-2.89
AMPD	AMP deaminase	1.17
AS	Hydroquinone glucosyltransferase	-1.50
ASAT1	Acyl-CoA--sterol O-acyltransferase 1	-3.88
At1g08940	Phosphoglycerate mutase-like protein AT74H	4.44
At1g30700	Berberine bridge enzyme-like 8	2.40
At2g26230	Uricase	3.25
At3g03980	NADPH-dependent aldehyde reductase-like protein, chloroplastic	3.38
At3g16150	Probable isoaspartyl peptidase/L-asparaginase 2	3.18
At3g50280	Uncharacterized acetyltransferase At3g50280	-1.07
At4g17486	DeSI-like protein At4g17486	9.99
At4g20840	Berberine bridge enzyme-like 21	-2.87
At4g33920	Probable protein phosphatase 2C 63	4.12
At5g06060	Tropinone reductase homolog At5g06060	-2.85
At5g07800	Flavin-containing monooxygenase FMO GS-OX-like 9	1.20
At5g08100	Isoaspartyl peptidase/L-asparaginase	3.44
At5g34940	Heparanase-like protein 3	2.35
At5g61250	Heparanase-like protein 2	2.67
At5g37990	Probable S-adenosylmethionine-dependent methyltransferase At5g37990	-1.49
BFRUCT3	Acid beta-fructofuranosidase 3, vacuolar	2.80
BH0283	Uncharacterized isomerase BH0283	2.86
BTG-26	Aldehyde dehydrogenase family 7 member A1	3.11
CCT1	Choline-phosphate cytidylyltransferase 1	4.07
CDA1	Cytidine deaminase 1	1.23
CEQORH	Chloroplast envelope quinone oxidoreductase homolog	1.08
CER1	Very-long-chain aldehyde decarbonylase CER1	5.86
clpP	ATP-dependent Clp protease proteolytic subunit	-9.28
COAE	Dephospho-CoA kinase	1.07
CST3	Cystatin	9.58
CST3	Cystatin-C	-10.53
CXE17	Probable carboxylesterase 17	-1.96
DBR	2-alkenal reductase (NADP(+)-dependent)	2.28
CYP73A5	Trans-cinnamate 4-monooxygenase	2.13
CYP81E8	Cytochrome P450 81E8	1.55
CYP82A3	Cytochrome P450 82A3	-2.50
CYSEP	Vignain	1.32
DIM	Delta(24)-sterol reductase	1.17
DOG1	DELAY OF GERMINATION 1	1.93
F3H-3	Flavanone 3-dioxygenase 3	2.68

FXG1	Alpha-L-fucosidase 3	-3.11
GAPN	NADP-dependent glyceraldehyde-3-phosphate dehydrogenase	-1.18
Gba2	Non-lysosomal glucosylceramidase	1.08
GLCNAC1PUT1	UDP-N-acetylglucosamine diphosphorylase 1	1.37
GLCAT14A	Beta-glucuronosyltransferase GlcAT14A	-1.10
GLOX	Aldehyde oxidase GLOX	-1.46
GPX6	Probable phospholipid hydroperoxide glutathione peroxidase 6, mitochondrial	2.48
GRXC9	Glutaredoxin-C9	13.18
GT6	UDP-glucose flavonoid 3-O-glucosyltransferase 6	7.66
guaA	Probable GMP synthase [glutamine-hydrolyzing]	1.83
HAL3A	Phosphopantothenoylcysteine decarboxylase	2.33
Hdhd3	Haloacid dehalogenase-like hydrolase domain-containing protein 3	3.48
HPPR	Hydroxyphenylpyruvate reductase	4.39
hsp90ab1	Heat shock protein HSP 90-beta	10.04
HST	Shikimate O-hydroxycinnamoyltransferase	1.16
ICL	Isocitrate lyase	3.47
KTI2	Miraculin	-2.47
LARP1A	La-related protein 1A	4.26
LECRK1	G-type lectin S-receptor-like serine/threonine-protein kinase LECRK1	1.97
LGALS1	Galectin-1	-9.97
LGALS1	Beta-galactoside-binding lectin	10.96
LUG	Transcriptional corepressor LEUNIG	-1.74
LUH	Transcriptional corepressor LEUNIG_HOMOLOG	2.77
maa	Probable maltose O-acetyltransferase	1.75
MLS	Malate synthase, glyoxysomal	-5.23
NDB2	External alternative NAD(P)H-ubiquinone oxidoreductase B2, mitochondrial	-2.49
NUDT17	Nudix hydrolase 17, mitochondrial	-4.39
OMT3	Probable O-methyltransferase 3	-4.60
OsI_27030	1-Cys peroxiredoxin	-2.08
P2A12	F-box protein PP2-A12	-1.11
PDCB2	PLASMODESMATA CALLOSE-BINDING PROTEIN 2	1.14
PEC-2	Thioredoxin H-type	2.01
PER52	Peroxidase 52	-3.30
PLL5	Probable protein phosphatase 2C 4	-1.57
PTEN1	Phosphatidylinositol 3,4,5-trisphosphate 3-phosphatase and protein-tyrosine-phosphatase PTEN1	-1.46
PUX4	Plant UBX domain-containing protein 4	-1.78
Pyroxd2	Pyridine nucleotide-disulfide oxidoreductase domain-containing protein 2	2.24
PYROXD2	Pyridine nucleotide-disulfide oxidoreductase domain-containing protein 2	1.22
QCT	Glutaminyl-peptide cyclotransferase	1.17
RBL5	RHOMBOID-like protein 5	-1.70
RCOM_0699480	Glycosyltransferase family 92 protein RCOM_0530710	1.17
ROD1	Phosphatidylcholine:diacylglycerol cholinephosphotransferase 1	1.76
SBT1.3	Subtilisin-like protease SBT1.3	-3.11
SDH6	Succinate dehydrogenase subunit 6, mitochondrial	-6.11
SEN102	Thiol protease SEN102	2.06
SERPINB6	Serpin B6	10.35

SERPINH1	Serpin H1	-8.95
SQD1	UDP-sulfoquinovose synthase, chloroplastic	1.16
SSL10	Protein STRICTOSIDINE SYNTHASE-LIKE 10	1.61
STR17	Rhodanese-like domain-containing protein 17	2.81
TBL39	Protein trichome birefringence-like 39	1.43
TBL6	Protein trichome birefringence-like 6	-1.20
TKL-1	Transketolase-1, chloroplastic	8.92
TOGT1	Scopoletin glucosyltransferase	-1.21
TPP1	Thylakoidal processing peptidase 1, chloroplastic	3.33
UREG	Urease accessory protein G	2.92

Table S3. Transport genes differentially expressed in CR and CS.

Gene name	Protein name	log_2 Ratio (CR/CS)
**Transmembrane transport**		
ABCB19	ABC transporter B family member 19	4.07
ABCB2	ABC transporter B family member 2	-1.18
ABCB9	ABC transporter B family member 9	2.47
ABCC10	ABC transporter C family member 10	-1.79
ABCC3	ABC transporter C family member 3	-2.09
ABCF1	ABC transporter F family member 1	-1.25
ABCG1	ABC transporter G family member 1	1.14
ABCG15	ABC transporter G family member 15	2.03
ABCG31	ABC transporter G family member 31	4.35
ABCG35	ABC transporter G family member 35	-2.86
ABCG39	ABC transporter G family member 39	-1.33
ABCG39	ABC transporter G family member 39	-1.87
ABCG5	ABC transporter G family member 5	2.61
ABCG51	ABC transporter G family member 51	-1.59
ABCG9	ABC transporter G family member 9	1.64
ACA12	Calcium-transporting ATPase 12, plasma membrane-type	9.37
ACT1	Actin-1	1.80
ACTA1	Actin, alpha skeletal muscle	-11.81
actba	Actin, cytoplasmic 1	3.86
ACTG1	Actin, cytoplasmic 2	-3.46
ACTG2	Actin, alpha cardiac muscle 2	-5.96
AHA4	ATPase 4, plasma membrane-type	3.77
AHA6	ATPase 6, plasma membrane-type	-3.40
AHA9	ATPase 9, plasma membrane-type	-1.22
ANT1	ADP,ATP carrier protein 1, mitochondrial	1.10
At1g09380	WAT1-related protein At1g09380	8.96
At4g08290	WAT1-related protein At4g08290	-9.03
At4g08300	WAT1-related protein At4g08300	11.94
At5g47470	WAT1-related protein At5g47470	-1.77
At5g57480	AAA-ATPase At5g57480	2.76
BOR1	Boron transporter 1	1.87
BOR3	Probable boron transporter 3	2.04
BOR4	Boron transporter 4	2.28
BOR6	Probable boron transporter 6	4.20
DDB_G0281815	Golgi to ER traffic protein 4 homolog	1.33
DIT1	Dicarboxylate transporter 1, chloroplastic	-1.13
DTX25	Protein DETOXIFICATION 25	-1.96
DTX28	Protein DETOXIFICATION 28	-3.22
DTX29	Protein DETOXIFICATION 29	-1.33
DTX31	Protein DETOXIFICATION 31	-6.16
DTX33	Protein DETOXIFICATION 33	-1.07
DTX40	Protein DETOXIFICATION 40	-3.41
DTX42	Protein DETOXIFICATION 42	-1.04
DTX48	Protein DETOXIFICATION 48	3.40
DTX49	Protein DETOXIFICATION 49	-5.37
DTX55	Protein DETOXIFICATION 55	2.45

EXO84A	Exocyst complex component EXO84A	1.06
NDT1	Nicotinamide adenine dinucleotide transporter 1, chloroplastic	-8.15
PDR1	Pleiotropic drug resistance protein 1	-2.52
SAR1A	GTP-binding protein SAR1A	3.11
SAR1B	GTP-binding protein SAR1B	2.89
sec14	Sec14 cytosolic factor	2.34
Slc25a3	Phosphate carrier protein, mitochondrial	10.10
SLC25A3	Phosphate carrier protein, mitochondrial	8.86
SULTR3;4	Probable sulfate transporter 3.4	1.99
SULTR3;5	Probable sulfate transporter 3.5	-2.53
VTI11	Vesicle transport v-SNARE 11	4.51
VTI13	Vesicle transport v-SNARE 13	-2.38
**Carbohydrate transport**		
At1g67300	Probable plastidic glucose transporter 2	1.12
At1g75220	Sugar transporter ERD6-like 6	1.21
At1g78830	EP1-like glycoprotein 2	4.00
At1g78850	EP1-like glycoprotein 3	3.56
At4g32390	Probable sugar phosphate/phosphate translocator At4g32390	2.19
At5g11230	Probable sugar phosphate/phosphate translocator At5g11230	-4.17
EP1	Epidermis-specific secreted glycoprotein EP1	-5.42
ERD6	Sugar transporter ERD6	2.47
EULS3	Ricin B-like lectin EULS3	4.42
MST1	Sugar transport protein MST1	-1.09
PLT5	Polyol transporter 5	-2.31
PLT6	Probable polyol transporter 6	-1.92
slc37a2	Glucose-6-phosphate exchanger SLC37A2	-3.84
slc37a2	Glucose-6-phosphate exchanger SLC37A2	3.05
Slc37a2	Glucose-6-phosphate exchanger SLC37A2	-1.13
STP1	Sugar transport protein 1	8.84
STP12	Sugar transport protein 12	5.61
STP13	Sugar transport protein 13	3.28
STP5	Sugar transport protein 5	-1.29
SWEET12	Bidirectional sugar transporter SWEET12	-2.05
SWEET14	Bidirectional sugar transporter SWEET14	1.57
SWEET15	Bidirectional sugar transporter SWEET15	9.57
SWEET5	Bidirectional sugar transporter SWEET5	2.05
UXT2	UDP-xylose transporter 2	1.71
UXT3	UDP-xylose transporter 3	-1.82
**lipid transport**		
N/A	Non-specific lipid-transfer protein	2.88
AATL1	Lysine histidine transporter-like 8	3.11
ABCA2	ABC transporter A family member 2	-3.19
ABCA7	ABC transporter A family member 7	-1.76
ALA2	Phospholipid-transporting ATPase 2	1.91
ALIS1	ALA-interacting subunit 1	4.45
LTP10	Non-specific lipid-transfer protein 3	3.41
LTPG1	Non-specific lipid transfer protein GPI-anchored 1	3.17
MALD3	Non-specific lipid-transfer protein	4.55
PATL4	Patellin-4	2.03
PEX13	Peroxisomal membrane protein 13	8.74
PEX6	Peroxisome biogenesis protein 6	-1.94

SCA	Non-specific lipid-transfer protein	2.52
SYT4	Synaptotagmin-4	1.38
SYT5	Synaptotagmin-5	3.27
**Amino acid transport**		
AAP2	Amino acid permease 2	2.54
AAP3	Amino acid permease 3	-1.06
ANT1	Amino acid transporter ANT1	4.49
At1g25530	Lysine histidine transporter-like 6	9.12
At2g39510	WAT1-related protein At2g39510	3.55
AVT1H	Amino acid transporter AVT1H	-1.23
AVT6A	Amino acid transporter AVT6A	-1.05
CAT1	Cationic amino acid transporter 1	1.93
CAT7	Cationic amino acid transporter 7, chloroplastic	1.37
LHT1	Lysine histidine transporter 1	-4.53
YPQ1	Probable vacuolar amino acid transporter YPQ1	-1.18
**Protein transport**		
14-3-3zeta	14-3-3 protein zeta	9.50
AHB1	Non-symbiotic hemoglobin 1	3.40
ahsa	Activator of 90 kDa heat shock protein ATPase homolog	3.83
AKR2A	Ankyrin repeat domain-containing protein 2A	3.44
AP2M1	AP-2 complex subunit mu	9.22
AP3BA	AP3-complex subunit beta-A	-8.68
ARF2-A	ADP-ribosylation factor 2-A	3.67
ASNAP2	Alpha-soluble NSF attachment protein 2	2.47
At1g21900	Transmembrane emp24 domain-containing protein p24delta5	2.75
At3g09030	BTB/POZ domain-containing protein At3g09030	-1.01
At3g13860	Chaperonin CPN60-like 2, mitochondrial	1.04
At5g01750	Protein LURP-one-related 15	3.29
ATG1C	Serine/threonine-protein kinase ATG1c	1.01
ATG8F	Autophagy-related protein 8f	-1.02
CHC1	Clathrin heavy chain 1	3.25
CLC2	Clathrin light chain 2	2.99
GF14B	14-3-3-like protein B	1.15
GF14C	14-3-3-like protein GF14-C	-3.76
GRF12	14-3-3-like protein GF14 iota	1.16
HSPB1	Heat shock protein beta-1	-10.39
IMPA1	Importin subunit alpha-1	1.22
KEU	SNARE-interacting protein KEULE	3.38
KN	Syntaxin-related protein KNOLLE	3.06
MON1	Vacuolar fusion protein MON1 homolog	-1.06
MSBP2	Membrane steroid-binding protein 2	-1.46
naca	Nascent polypeptide-associated complex subunit alpha	10.66
NACA	Nascent polypeptide-associated complex subunit alpha	-7.51
NBR1	Protein NBR1 homolog	5.11
NTF2A	Nuclear transport factor 2A	2.09
NTF2B	Nuclear transport factor 2B	2.39
OEP16	Outer envelope pore protein 16, chloroplastic	-2.06
OEP163	Outer envelope pore protein 16-3, chloroplastic/mitochondrial	8.32
Os12g0104800	Clathrin heavy chain 2	8.71
PAT05	Probable protein S-acyltransferase 5	8.75
PAT18	Protein S-acyltransferase 18	2.06

RAB7	Ras-related protein Rab7	-1.72
RABA1F	Ras-related protein RABA1f	1.81
RABA4A	Ras-related protein RABA4a	8.15
RABA6A	Ras-related protein RABA6a	4.51
RABB1C	Ras-related protein RABB1c	3.18
RABD2C	Ras-related protein RABD2c	2.58
RABF2A	Ras-related protein RABF2a	7.93
RABG3D	Ras-related protein RABG3d	-1.56
RABG3F	Ras-related protein RABG3f	2.81
RBK2	Receptor-like cytosolic serine/threonine-protein kinase RBK2	2.34
RHN1	Ras-related protein RHN1	-1.05
SEC1A	Protein transport Sec1a	5.33
sec61a	Protein transport protein Sec61 subunit alpha	3.03
SEC61B	Protein transport protein Sec61 subunit beta	8.93
SYP112	Syntaxin-112	3.04
SYP121	Syntaxin-121	8.61
SYP122	Syntaxin-122	5.21
TIC214	Protein TIC 214	-3.46
TIC32	Short-chain dehydrogenase TIC 32, chloroplastic	-2.56
TMN11	Transmembrane 9 superfamily member 11	8.31
TOL6	TOM1-like protein 6	8.72
TOL9	TOM1-like protein 9	1.00
TOM9-2	Mitochondrial import receptor subunit TOM9-2	1.18
VAMP711	Vesicle-associated membrane protein 711	4.60
VAMP722	Vesicle-associated membrane protein 722	2.37
VAMP727	Vesicle-associated membrane protein 727	-1.26
ywhaba	14-3-3 protein beta/alpha-A	9.70
VPS2.2	Vacuolar protein sorting-associated protein 2 homolog 2	1.02
VPS32.1	Vacuolar protein sorting-associated protein 32 homolog 1	2.63
VSR1	Vacuolar-sorting receptor 1	3.13
**Metal ion transport**		
N/A	Stellacyanin	-2.59
ABCG36	ABC transporter G family member 36	3.02
AGD14	Probable ADP-ribosylation factor GTPase-activating protein AGD14	2.08
AKT1	Potassium channel AKT1	-1.70
AKT6	Potassium channel AKT6	3.50
AOP1	Probable 2-oxoglutarate-dependent dioxygenase AOP1	-2.19
AOP1.2	Probable 2-oxoglutarate-dependent dioxygenase AOP1.2	-4.50
At1g06620	1-aminocyclopropane-1-carboxylate oxidase homolog 1	-1.01
At1g73020	Anoctamin-like protein At1g73020	1.07
At2g01810	PHD finger protein At2g01810	-1.56
At2g44790	Uclacyanin-2	-9.23
At3g21360	Clavaminate synthase-like protein At3g21360	1.04
At3g43660	Vacuolar iron transporter homolog 4	3.51
At3g54510	CSC1-like protein At3g54510	1.32
BCB	Umecyanin	2.80
Bp10	L-ascorbate oxidase homolog	-3.63
CALR	Calreticulin	-9.70
CAT2	Catalase isozyme 2	-1.07
CAX3	Vacuolar cation/proton exchanger 3	-2.15
CBSDUF1	DUF21 domain-containing protein At4g14240	-1.10

CCS	Copper chaperone for superoxide dismutase, chloroplastic/cytosolic	3.18
CHX15	Cation/H(+) antiporter 15	1.39
CHX4	Cation/H(+) antiporter 4	1.41
CML3	Calmodulin-like protein 3	1.13
CML35	Probable calcium-binding protein CML35	3.55
CML50	Probable calcium-binding protein CML50	3.93
CNGC2	Cyclic nucleotide-gated ion channel 2	2.52
CNX1	Calnexin homolog 1	2.68
CYB561D	Probable transmembrane ascorbate ferrireductase 4	2.07
CYP79A2	Phenylalanine N-monooxygenase	2.08
DRT112	Plastocyanin major isoform, chloroplastic	10.97
ETFQO	Electron transfer flavoprotein-ubiquinone oxidoreductase, mitochondrial	8.56
fcpA	Probable C-terminal domain small phosphatase	1.49
FLS	Flavonol synthase/flavanone 3-hydroxylase	9.70
FSD1	Superoxide dismutase [Fe] 1, chloroplastic	1.80
FTH	Ferritin, heavy subunit	9.85
FTH	Ferritin, middle subunit	11.47
FTH1	Ferritin heavy chain	-3.56
FTL	Ferritin light chain	-10.85
Ftl1	Ferritin light chain 1	-10.43
GCL2	LanC-like protein GCL2	1.08
gp63	Leishmanolysin homolog	4.80
GRXS7	Monothiol glutaredoxin-S7, chloroplastic	-1.20
HAK13	Probable potassium transporter 13	-1.13
HB2	Non-symbiotic hemoglobin 2	1.39
HIPP05	Heavy metal-associated isoprenylated plant protein 5	3.94
HIPP06	Heavy metal-associated isoprenylated plant protein 6	2.28
HIPP09	Heavy metal-associated isoprenylated plant protein 9	3.31
HIPP21	Heavy metal-associated isoprenylated plant protein 21	-1.29
HIPP23	Heavy metal-associated isoprenylated plant protein 23	1.70
HIPP26	Heavy metal-associated isoprenylated plant protein 26	-1.85
HIPP32	Heavy metal-associated isoprenylated plant protein 32	1.25
HIPP37	Heavy metal-associated isoprenylated plant protein 37	1.89
HIPP39	Heavy metal-associated isoprenylated plant protein 39	-2.02
IQD1	Protein IQ-DOMAIN 1	1.43
IRT2	Fe(2+) transport protein 2	6.57
KAT3	Potassium channel KAT3	2.70
KEA4	K(+) efflux antiporter 4	1.03
LAC14	Laccase-14	-2.63
LAC6	Laccase-6	2.00
LAC7	Laccase-7	-3.26
Lonrf3	LON peptidase N-terminal domain and RING finger protein 3	2.77
LSC30	Ferritin-1, chloroplastic	1.58
LTP1	Non-specific lipid-transfer protein 1	10.09
mcfB	Mitochondrial substrate carrier family protein B	-1.01
mgtA	Magnesium-transporting ATPase, P-type 1	-1.29
MHB1	Non-symbiotic hemoglobin 1	-2.02
MICU	Calcium uptake protein, mitochondrial	3.51
MT3	Metallothionein-like protein type 3	4.82
MT4B	Metallothionein-like protein 4B	4.11

MTP4	Metal tolerance protein 4	-2.51
NAKR2	Protein SODIUM POTASSIUM ROOT DEFECTIVE 2	7.85
NRAMP3	Metal transporter Nramp3	-1.24
NRAMP6	Metal transporter Nramp6	-1.23
NUDT16	Nudix hydrolase 16, mitochondrial	1.35
NUDT21	Nudix hydrolase 21, chloroplastic	4.97
OBE3	Protein OBERON 3	3.28
OPT3	Oligopeptide transporter 3	-2.05
PAP18	Purple acid phosphatase 18	5.17
PAP2	Purple acid phosphatase 2	1.56
PAP27	Probable inactive purple acid phosphatase 27	1.07
pdcA	Pyruvate decarboxylase	2.09
PETE	Plastocyanin, chloroplastic	3.41
PETF	Ferredoxin	9.83
NHX2	Sodium/hydrogen exchanger 2	1.78
NIP1	NEP1-interacting protein 1	8.72
pKIWI504	Metallothionein-like protein type 2	-1.47
PSBQ1	Oxygen-evolving enhancer protein 3-1, chloroplastic	10.15
PSBQ2	Oxygen-evolving enhancer protein 3-2, chloroplastic	9.40
Pvalb	Parvalbumin alpha	-10.53
S100A4	Ictacalcin	11.55
Sftpa1	Pulmonary surfactant-associated protein A	-9.09
SKU5	Monocopper oxidase-like protein SKU5	1.16
SOP1	Peroxygenase	-5.35
SPARC	SPARC	-7.27
THBS1	Thrombospondin-1	-12.10
VIT_05s0020g01830	CASP-like protein 1D1	-2.25
VIT_07s0104g01350	CASP-like protein 1E1	5.55
VIT_09s0002g03780	CASP-like protein 3A1	1.23
VIT_14s0068g01400	CASP-like protein 1F2	2.15
ZIFL1	Protein ZINC INDUCED FACILITATOR-LIKE 1	-2.24
ZIP10	Probable zinc transporter 10	-8.62
ZIP3	Zinc transporter 3	-2.24
**Others**		
7-Oct	Organic cation/carnitine transporter 7	-1.37
N/A	Pathogen-related protein	1.25
N/A	Polyprotein P3	2.97
N/A	Probable cytochrome c biosynthesis protein	12.80
N/A	RNA2 polyprotein	-13.34
ALMT10	Aluminum-activated malate transporter 10	-2.19
ALMT12	Aluminum-activated malate transporter 12	-9.80
ALMT13	Aluminum-activated malate transporter 13	-10.99
arv1	Protein arv1 homolog	1.16
At1g02270	Uncharacterized calcium-binding protein At1g02270	4.18
At2g25060	Early nodulin-like protein 1	11.75
At3g01520	Universal stress protein A-like protein	9.24
At3g28850	Uncharacterized protein At3g28850	2.94
At4g26020	Protein At-4/1	1.82
At4g27520	Early nodulin-like protein 2	3.27
At5g25090	Early nodulin-like protein 3	2.97
At5g39570	Uncharacterized protein At5g39570	2.49

At5g39865	Uncharacterized protein At5g39865	2.14
ATP5MC3	ATP synthase F(0) complex subunit C3, mitochondrial	10.17
ATPA	ATP synthase subunit alpha, mitochondrial	3.74
AVP1	Pyrophosphate-energized vacuolar membrane proton pump 1	9.37
AZG2	Adenine/guanine permease AZG2	-1.30
BAC2	Mitochondrial arginine transporter BAC2	-1.64
CD9	CD9 antigen	9.66
CIPK6	CBL-interacting serine/threonine-protein kinase 6	3.31
CPR	NADPH--cytochrome P450 reductase	-1.66
CURT1B	Protein CURVATURE THYLAKOID 1B, chloroplastic	9.07
CXXS1	Thioredoxin-like protein CXXS1	8.75
ECH	Golgi apparatus membrane protein-like protein ECHIDNA	-1.79
ENODL1	Early nodulin-like protein 1	2.12
ENT3	Equilibrative nucleotide transporter 3	1.52
ETFB	Electron transfer flavoprotein subunit beta, mitochondrial	1.70
FATB1	Palmitoyl-acyl carrier protein thioesterase, chloroplastic	1.43
FH11	Formin-like protein 11	1.02
GRXC3	Glutaredoxin-C3	2.92
GRXC6	Glutaredoxin-C6	2.48
INT1	Inositol transporter 1	-1.05
INT2	Probable inositol transporter 2	-2.45
MBD10	Methyl-CpG-binding domain-containing protein 10	2.88
MSL10	Mechanosensitive ion channel protein 10	1.21
Mtatp6	ATP synthase subunit a	-12.08
MT-ATP6	ATP synthase subunit a	-10.24
Mtnd2	NADH-ubiquinone oxidoreductase chain 2	-10.29
MT-ND2	NADH-ubiquinone oxidoreductase chain 2	11.58
Mtnd4	NADH-ubiquinone oxidoreductase chain 4	-10.50
MT-ND4L	NADH-ubiquinone oxidoreductase chain 4L	-10.33
Mtnd5	NADH-ubiquinone oxidoreductase chain 5	-9.45
MT-ND5	NADH-ubiquinone oxidoreductase chain 5	-10.20
NIK1	Protein NSP-INTERACTING KINASE 1	2.45
NPF1.2	Protein NRT1/ PTR FAMILY 1.2	-4.17
NPF2.11	Protein NRT1/ PTR FAMILY 2.11	-1.03
NPF2.13	Protein NRT1/ PTR FAMILY 2.13	-2.59
NPF5.2	Protein NRT1/ PTR FAMILY 5.2	1.44
NPF5.6	Protein NRT1/ PTR FAMILY 5.6	-3.92
NPF6.3	Protein NRT1/ PTR FAMILY 6.3	2.95
NPF6.4	Protein NRT1/ PTR FAMILY 6.4	2.37
NRG2	Nitrate regulatory gene2 protein	3.46
NRT3.1	High-affinity nitrate transporter 3.1	-3.87
PHO1;H3	Phosphate transporter PHO1 homolog 3	-1.39
PHO1-1	Phosphate transporter PHO1-1	2.82
PHO1-H1	Phosphate transporter PHO1 homolog 1	3.17
PHO1-H5	Phosphate transporter PHO1 homolog 5	-1.57
pho-5	Repressible high-affinity phosphate permease	9.08
PHT1-11	Inorganic phosphate transporter 1-11	3.50
PHT1-4	Inorganic phosphate transporter 1-4	1.53
PILS2	Protein PIN-LIKES 2	-4.37
PIN1A	Auxin efflux carrier component 1a	2.08
PIN3	Auxin efflux carrier component 3	2.03

PIN8	Auxin efflux carrier component 8	3.32
PIP1-3	Aquaporin PIP1-3	1.12
PIP2-7	Aquaporin PIP2-7	1.98
PIP5K6	Phosphatidylinositol 4-phosphate 5-kinase 6	1.53
PMEI10	21 kDa protein	3.26
PRA1B4	PRA1 family protein B4	3.22
PRA1F2	PRA1 family protein F2	1.20
PRDX1	Peroxiredoxin	9.24
PUP11	Probable purine permease 11	1.67
PUP14	Purine permease 14	4.23
rraA	Regulator of ribonuclease activity A	2.78
Sftpc	Pulmonary surfactant-associated protein C	-11.64
SIP1-1	Aquaporin SIP1-1	-1.32
SLC25A5	ADP/ATP translocase 2	11.15
TBL19	Protein trichome birefringence-like 19	1.24
TDX	TPR repeat-containing thioredoxin TDX	1.39
WDR44	WD repeat-containing protein 44	1.48
ycf2	Protein Ycf2	-2.66
ycf2-A	Protein Ycf2	-1.16
ycf2-A	Protein Ycf2	4.40
ycf2-A	Protein Ycf2	-2.83
ycf2-A	Protein Ycf2	-5.24
TIP1-3	Aquaporin TIP1-3	-2.84
TIP2-1	Aquaporin TIP2-1	5.04
TUBB1	Tubulin beta-1 chain	2.05
VHA-d2	V-type proton ATPase subunit d2	-1.91

Table S4. Transcription genes factor differentially expressed in CR and CS.

Gene name	Protein name	log_2 Ratio (CR/CS)
**Transcription factors**		
1-Sep	Developmental protein SEPALLATA 1	8.74
AFP3	Ninja-family protein AFP3	1.21
AG1	Floral homeotic protein AGAMOUS	9.61
AG2	Floral homeotic protein AGAMOUS	7.24
AGL15	Agamous-like MADS-box protein AGL15	3.63
AHL1	AT-hook motif nuclear-localized protein 1	1.68
AHL10	AT-hook motif nuclear-localized protein 10	1.14
AHL17	AT-hook motif nuclear-localized protein 17	-2.51
AHL23	AT-hook motif nuclear-localized protein 23	-2.60
AHL26	AT-hook motif nuclear-localized protein 26	-2.45
AHL5	AT-hook motif nuclear-localized protein 5	1.76
AIL1	AP2-like ethylene-responsive transcription factor AIL1	4.64
AIL6	AP2-like ethylene-responsive transcription factor AIL6	3.14
AMS	Transcription factor ABORTED MICROSPORES	6.25
ANL2	Homeobox-leucine zipper protein ANTHOCYANINLESS 2	2.95
ANT	AP2-like ethylene-responsive transcription factor ANT	2.08
AP1	Floral homeotic protein APETALA 1	5.10
AP1	Floral homeotic protein APETALA 1	8.17
AP2	Floral homeotic protein APETALA 2	1.74
ARF17	Auxin response factor 17	1.41
ARF31	B3 domain-containing protein At2g36080	1.44
ARF8	Auxin response factor 8	1.29
At5g14285	B3 domain-containing protein At2g36080	10.29
At5g42700	B3 domain-containing protein At5g42700	2.51
ATHB-12	Homeobox-leucine zipper protein ATHB-12	5.89
ATHB-20	Homeobox-leucine zipper protein ATHB-20	3.93
ATHB-40	Homeobox-leucine zipper protein ATHB-40	1.38
ATHB-6	Homeobox-leucine zipper protein ATHB-6	2.11
BEH2	BES1/BZR1 homolog protein 2	2.51
BEL1	Homeobox protein BEL1 homolog	-2.09
BHLH11	Transcription factor bHLH11	3.42
BHLH117	Transcription factor bHLH117	4.30
BHLH30	Transcription factor bHLH30	1.76
BHLH48	Transcription factor bHLH48	2.24
BHLH49	Transcription factor bHLH49	1.12
BHLH62	Transcription factor bHLH62	1.23
BHLH63	Transcription factor bHLH63	1.49
BHLH66	Transcription factor bHLH66	2.74
BHLH67	Transcription factor bHLH67	1.07
BHLH68	Transcription factor bHLH68	-1.90
BHLH74	Transcription factor bHLH74	1.13
BHLH91	Transcription factor bHLH91	7.59
BHLH94	Transcription factor bHLH94	-1.69
BLH11	BEL1-like homeodomain protein 11	-1.34
BLH7	BEL1-like homeodomain protein 7	-1.10
BPE	Transcription factor BPE	2.71

BTF3	Transcription factor BTF3	10.25
BZIP02	Basic leucine zipper 2	2.08
BZIP1	Basic leucine zipper 1	8.65
BZIP2	bZIP transcription factor 2	1.58
BZIP25	Basic leucine zipper 25	5.21
BZIP53	bZIP transcription factor 53	2.97
BZIP60	bZIP transcription factor 60	4.24
C1	Anthocyanin regulatory C1 protein	-4.13
CDC5	Cell division cycle 5-like protein	8.91
CDF2	Cyclic dof factor 2	-1.94
CSA	Transcription factor CSA	-1.11
DEFA	Floral homeotic protein DEFICIENS	2.16
DIVARICATA	Transcription factor DIVARICATA	1.16
DOGL2	Protein DOG1-like 2	1.77
DOGL3	Protein DOG1-like 3	-1.59
DREB2D	Dehydration-responsive element-binding protein 2D	-3.81
DREB2E	Dehydration-responsive element-binding protein 2E	3.79
DREB2G	Dehydration-responsive element-binding protein 2G	1.38
DREB3	Dehydration-responsive element-binding protein 3	2.39
DYT1	Transcription factor DYT1	8.78
EJ2	MADS-box protein EJ2	8.23
ERF1A	Ethylene-responsive transcription factor 1A	-3.96
FAMA	Transcription factor FAMA	9.21
FL	Floricaula/leafy homolog	10.03
FRS3	Protein FAR1-RELATED SEQUENCE 3	-1.51
FRS5	Protein FAR1-RELATED SEQUENCE 5	1.64
FRS7	Protein FAR1-RELATED SEQUENCE 7	-8.25
FRS8	Protein FAR1-RELATED SEQUENCE 8	1.69
GATA12	GATA transcription factor 12	3.44
GATA4	GATA transcription factor 4	1.78
GATA5	GATA transcription factor 5	1.34
GATA8	GATA transcription factor 8	3.63
GATA9	GATA transcription factor 9	1.84
GEBPL	GLABROUS1 enhancer-binding protein-like	3.75
GL2	Homeobox-leucine zipper protein GLABRA 2	-1.39
GT-2	Trihelix transcription factor GT-2	-1.16
GT-3B	Trihelix transcription factor GT-3b	-3.71
GT-4	Trihelix transcription factor GT-4	-1.13
GTE1	Transcription factor GTE1	-1.81
gtf2f2	General transcription factor IIF subunit 2	-1.27
HAT14	Homeobox-leucine zipper protein HAT14	1.34
HD1	Homeobox protein HD1	-1.17
HDG12	Homeobox-leucine zipper protein HDG12	-1.87
HHO2	Transcription factor HHO2	8.51
HMGB9	High mobility group B protein 9	1.70
HOX27	Homeobox-leucine zipper protein HOX27	2.87
HSF24	Heat shock factor protein HSF24	-1.18
HSFB1	Heat stress transcription factor B-1	2.21
HSFB2C	Heat stress transcription factor B-2c	-1.02
HSFB4	Heat stress transcription factor B-4	-2.21
HSFC1	Heat stress transcription factor C-1	6.15

IBL1	Transcription factor IBH1-like 1	1.51
ID3	DNA-binding protein inhibitor ID-3	-9.63
KAN2	Probable transcription factor KAN2	1.90
KAN4	Probable transcription factor KAN4	2.51
KN1	Homeobox protein knotted-1-like 2	1.18
KN1	Homeotic protein knotted-1	1.24
KNAT3	Homeobox protein knotted-1-like 3	-1.09
LBD20	LOB domain-containing protein 20	1.52
LBD29	LOB domain-containing protein 29	2.08
LBD41	LOB domain-containing protein 41	3.62
LHW	Transcription factor LHW	1.07
LSH10	Protein LIGHT-DEPENDENT SHORT HYPOCOTYLS 10	-4.47
LSH3	Protein LIGHT-DEPENDENT SHORT HYPOCOTYLS 3	2.20
LSH4	Protein LIGHT-DEPENDENT SHORT HYPOCOTYLS 4	-2.37
MADS3	MADS-box transcription factor 3	8.36
MADS57	MADS-box transcription factor 57	-3.06
MBF1B	Multiprotein-bridging factor 1b	2.51
MBF1C	Multiprotein-bridging factor 1c	1.11
MIP1B	B-box domain protein 31	-1.33
MtrDRAFT_AC149210g4v1	Probable histone H2A.2	2.46
MUTE	Transcription factor MUTE	-1.79
MYB105	Transcription factor MYB105	3.12
MYB14	Transcription factor MYB14	-5.76
MYB17	Transcription factor MYB17	2.06
MYB23	Transcription factor MYB23	-2.90
MYB25	Transcription factor MYB25	-1.90
MYB306	Myb-related protein 306	1.18
MYB308	Myb-related protein 308	-2.63
MYB330	Myb-related protein 330	-2.01
MYB35	Transcription factor MYB35	7.84
MYB36	Transcription factor MYB36	-4.45
MYB44	Transcription factor MYB44	-1.21
MYB46	Transcription factor MYB46	1.60
MYB61	Transcription factor MYB61	2.56
MYB77	Transcription factor MYB77	1.68
MYB8	Transcription factor MYB8	-2.24
MYBC1	Transcription factor MYBC1	1.30
MYR2	Myb-related protein 2	-1.07
NAC035	NAC domain-containing protein 35	1.26
NAC071	NAC domain-containing protein 71	1.72
NAC072	NAC domain-containing protein 72	-4.58
NAC075	NAC domain-containing protein 75	1.62
NAC078	NAC domain-containing protein 78	8.16
NAC083	NAC domain-containing protein 83	3.51
NAC087	NAC domain-containing protein 87	-2.50
NAC100	NAC domain-containing protein 100	-2.04
NAC104	NAC domain-containing protein 104	-9.16
NAC69	NAC domain-containing protein 69	2.33
NEN4	NEN4	-1.33
NFYA8	Nuclear transcription factor Y subunit A-8	1.83

NFYB6	Nuclear transcription factor Y subunit B-6	1.58
NIR1	Ferredoxin--nitrite reductase, chloroplastic	-4.52
NLP2	Protein NLP2	1.18
NRPB11	DNA-directed RNA polymerases II, IV and V subunit 11	-3.35
NRPB6A	DNA-directed RNA polymerases II, IV and V subunit 6A	3.05
NUCL1	Nucleolin 1	4.21
OFP13	Transcription repressor OFP13	3.29
OFP14	Transcription repressor OFP14	-1.70
OFP16	Transcription repressor OFP16	1.42
OFP3	Transcription repressor OFP3	1.09
OFP7	Transcription repressor OFP7	1.15
OFP8	Transcription repressor OFP8	8.96
PAN	Transcription factor PERIANTHIA	1.77
PIF4	Transcription factor PIF4	-1.98
PMADS2	Floral homeotic protein PMADS 2	13.38
PRE5	Transcription factor PRE5	5.11
RAP2-4	Ethylene-responsive transcription factor RAP2-4	2.92
RDR1	RNA-dependent RNA polymerase 1	1.09
RDR2	RNA-dependent RNA polymerase 2	1.60
SAP	Transcriptional regulator STERILE APETALA	-2.65
SCL18	Scarecrow-like protein 18	8.61
SCL3	Scarecrow-like protein 3	-2.25
SCL5	Scarecrow-like protein 5	4.16
SCRM	Transcription factor ICE1	2.15
SHI	Protein SHORT INTERNODES	4.51
SHR	Protein SHORT-ROOT	2.47
SIR	Assimilatory sulfite reductase (ferredoxin), chloroplastic	2.15
SIR1	Sulfite reductase 1 [ferredoxin], chloroplastic	-1.00
SPL6	Squamosa promoter-binding-like protein 6	-1.24
SPL9	Squamosa promoter-binding-like protein 9	-1.92
STOP1	Protein SENSITIVE TO PROTON RHIZOTOXICITY 1	1.77
TAF10	Transcription initiation factor TFIID subunit 10	4.47
TAGLN3	Transgelin-3	8.69
TCP15	Transcription factor TCP15	1.29
TCP20	Transcription factor TCP20	3.21
TCP4	Transcription factor TCP4	2.66
TCX2	tesmin/TSO1-like CXC 2	1.08
TFIIB1	Transcription initiation factor IIB-1	5.71
TGA10	bZIP transcription factor TGA10	7.03
TPR4	Topless-related protein 4	11.93
TRY	Transcription factor TRY	-1.35
TULP1	Tubby-like F-box protein 1	1.26
WIN1	Ethylene-responsive transcription factor WIN1	-2.32
WRKY11	Probable WRKY transcription factor 11	3.69
WRKY12	Probable WRKY transcription factor 12	-2.93
WRKY24	WRKY transcription factor WRKY24	-2.56
WRKY48	Probable WRKY transcription factor 48	-2.76
WRKY6	WRKY transcription factor 6	2.72
WRKY65	Probable WRKY transcription factor 65	-2.50
WRKY75	Probable WRKY transcription factor 75	9.10
**Zinc finger protein**		

At1g19860	Zinc finger CCCH domain-containing protein 6	-1.09
At1g66810	Zinc finger CCCH domain-containing protein 14	-1.04
At1g68200	Zinc finger CCCH domain-containing protein 15	1.28
At2g19810	Zinc finger CCCH domain-containing protein 20	4.03
At2g20280	Zinc finger CCCH domain-containing protein 21	-1.62
At2g25900	Zinc finger CCCH domain-containing protein 23	1.58
At2g40140	Zinc finger CCCH domain-containing protein 29	-1.58
AZF1	Zinc finger protein AZF1	-1.66
COL2	Zinc finger protein CONSTANS-LIKE 2	3.24
DOF3.5	Dof zinc finger protein DOF3.5	2.97
DOF3.6	Dof zinc finger protein DOF3.6	1.75
DOF5.4	Dof zinc finger protein DOF5.4	1.08
DOF5.7	Dof zinc finger protein DOF5.7	3.18
HAT	Zinc finger BED domain-containing protein DAYSLEEPER	7.47
JAG	Zinc finger protein JAGGED	11.05
KNU	Zinc finger protein KNUCKLES	10.30
MGP	Zinc finger protein MAGPIE	2.84
Os01g0518400	Zinc finger BED domain-containing protein RICESLEEPER 4	-4.86
Os03g0733400	Zinc finger BED domain-containing protein RICESLEEPER 2	-10.07
Os05g0239150	Zinc finger BED domain-containing protein RICESLEEPER 1	-5.40
Os05g0583200	Zinc finger BED domain-containing protein RICESLEEPER 3	-6.09
Os07g0682400	Zinc finger CCCH domain-containing protein 53	1.75
Os10g0391300	Zinc finger CCCH domain-containing protein 62	2.35
SAP12	Zinc finger AN1 domain-containing stress-associated protein 12	3.08
SAP2	Zinc finger A20 and AN1 domain-containing stress-associated protein 2	3.24
SAP4	Zinc finger A20 and AN1 domain-containing stress-associated protein 4	3.29
SAP5	Zinc finger A20 and AN1 domain-containing stress-associated protein 5	4.12
SAP7	Zinc finger A20 and AN1 domain-containing stress-associated protein 7	2.29
WIP2	Zinc finger protein WIP2	10.96
WIP4	Zinc finger protein WIP4	8.26
ZAT12	Zinc finger protein ZAT12	4.37
ZAT3	Zinc finger protein ZAT3	-9.35
ZAT5	Zinc finger protein ZAT5	-1.95
ZAT6	Zinc finger protein ZAT6	3.67
ZAT9	Zinc finger protein ZAT9	1.54
ZFP1	Zinc finger protein 1	-1.04
ZFP10	Zinc finger protein 10	9.95
ZFP3	Zinc finger protein 3	-1.11
ZHD1	Zinc-finger homeodomain protein 1	5.73
ZHD4	Zinc-finger homeodomain protein 4	7.49
ZHD8	Zinc-finger homeodomain protein 8	1.28
ZMYM1	Zinc finger MYM-type protein 1	-1.43
ZNHIT3	Zinc finger HIT domain-containing protein 3	2.51

**Reverse transcription**		
N/A	DNA polymerase	-1.05
N/A	Retrovirus-related Pol polyprotein from transposon TNT 1-94	12.95
N/A	Retrovirus-related Pol polyprotein from type-1 retrotransposable element R2	-3.47
N/A	Retrovirus-related Pol polyprotein from type-1 retrotransposable element R2	13.47
N/A	Retrovirus-related Pol polyprotein from type-1 retrotransposable element R2	10.44
N/A	RNA-directed RNA polymerase	-1.31
At4g24790	STICHEL-like 2	1.03
pol	Retrovirus-related Pol polyprotein from transposon 17.6	11.05
pol	Retrovirus-related Pol polyprotein from transposon 297	1.91
POL	Retrovirus-related Pol polyprotein from transposon 412	8.02
pol	Retrovirus-related Pol polyprotein from transposon gypsy	-6.21
pol	Retrovirus-related Pol polyprotein from transposon opus	3.73
pol	Retrovirus-related Pol polyprotein from type-2 retrotransposable element R2DM	-3.40
POLL	DNA polymerase lambda	1.36
RE1	Retrovirus-related Pol polyprotein from transposon RE1	-9.55
RE2	Retrovirus-related Pol polyprotein from transposon RE2	-9.60
TY3B-I	RNA-directed DNA polymerase homolog	-9.25
**Others**		
AGO1B	Protein argonaute 1B	3.18
alkB	Alpha-ketoglutarate-dependent dioxygenase AlkB	-1.00
ASI1	Protein ANTI-SILENCING 1	1.13
At1g07790	Histone H2B.1	1.68
At1g09620	Leucine--tRNA ligase, cytoplasmic	-2.34
At1g52740	Probable histone H2A variant 3	2.53
At1g65660	Pre-mRNA-splicing factor SLU7-A	2.60
At2g28720	Histone H2B.3	2.31
At2g30620	Histone H1	1.38
At3g19184	B3 domain-containing protein At3g19184	1.41
At3g58590	Pentatricopeptide repeat-containing protein At3g58590	1.42
At4g34730	Probable ribosome-binding factor A, chloroplastic	11.52
At5g02560	Histone H2A	1.16
ATXR6	Histone-lysine N-methyltransferase ATXR6	1.26
BARD1	BRCA1-associated RING domain protein 1	10.47
c12.1	Protein CWC15 homolog	2.33
CAD	Probable mannitol dehydrogenase	1.69
cdc45	Cell division control protein 45 homolog	1.52
CDC6	Cell division control protein 6 homolog	1.10
CDT1A	CDT1-like protein a, chloroplastic	1.48
CID11	Polyadenylate-binding protein-interacting protein 11	-1.23
cirbp-b	Cold-inducible RNA-binding protein B	9.70
CMT3	DNA (cytosine-5)-methyltransferase CMT3	1.35
CP29B	RNA-binding protein CP29B, chloroplastic	2.65
crop	Luc7-like protein	-1.13
CRS2A	Chloroplastic group IIB intron splicing facilitator CRS2-A, chloroplastic	1.00
CSN2	COP9 signalosome complex subunit 2	9.39

cwf17	Pre-mRNA-splicing factor cwf17	8.51
CXIP4	CAX-interacting protein 4	9.50
DDM1	ATP-dependent DNA helicase DDM1	6.67
DDX21	Nucleolar RNA helicase 2	9.81
Ddx50	ATP-dependent RNA helicase DDX50	9.17
DMC1	Meiotic recombination protein DMC1 homolog	2.13
DMT1	DNA (cytosine-5)-methyltransferase 1	1.27
DnaJ-1	DnaJ protein homolog 1	5.29
dpb4	DNA polymerase epsilon subunit D	1.05
dtl-a	Denticleless protein homolog A	1.62
EBP2	Probable rRNA-processing protein EBP2 homolog	1.21
EEF1D	Elongation factor 1-delta	9.76
EEF2	Elongation factor 2	-8.48
EMB2758	Pentatricopeptide repeat-containing protein At4g33990	1.30
eri2	ERI1 exoribonuclease 2	1.62
EXO1	Exonuclease 1	1.35
F46B6.12	UPF0587 protein F46B6.12	-4.53
Gins1	DNA replication complex GINS protein PSF1	1.07
GIP	Copia protein	10.24
GLO	Floral homeotic protein GLOBOSA	13.36
GRF3	Growth-regulating factor 3	2.37
GRF4	Growth-regulating factor 4	5.22
GRP10	Glycine-rich RNA-binding protein 10	3.23
GRXS13	Monothiol glutaredoxin-S13	4.43
H3-1.1	Histone H3.2	1.96
H3-7	Histone H3.2	2.98
HAC12	Histone acetyltransferase HAC12	1.59
HDT2	Histone deacetylase HDT2	2.98
HHT1	Histone H3.1	2.53
HIPP41	Heavy metal-associated isoprenylated plant protein 41	1.09
HIS2A	Histone H2AX	1.86
hmces	Abasic site processing protein HMCES	2.31
HMGB2	HMG1/2-like protein	1.54
HMGB2	HMG1/2-like protein	3.75
HMGIY2	HMG-Y-related protein A	1.45
K02A2.6	Uncharacterized protein K02A2.6	-1.53
KU70	ATP-dependent DNA helicase 2 subunit KU70	-1.49
L	RNA-directed RNA polymerase L	-8.80
LARP6A	La-related protein 6A	8.74
MAGO	mago nashi homolog	2.11
MCM3	DNA replication licensing factor MCM3	1.77
MCM5	DNA replication licensing factor MCM5	1.01
MCM9	Probable DNA helicase MCM9	1.59
MED37C	Probable mediator of RNA polymerase II transcription subunit 37c	5.15
MED6	Mediator of RNA polymerase II transcription subunit 6	-1.34
NAC081	Protein ATAF2	9.24
ORC1	Origin of replication complex subunit 1	2.34
ORC4	Origin of replication complex subunit 4	1.23
ORC6	Origin of replication complex subunit 6	1.37
ORTH2	E3 ubiquitin-protein ligase ORTHRUS 2	1.79

Os03g0120900	B3 domain-containing protein Os03g0120900	1.95
Os05g0235800	DNA replication licensing factor MCM6	1.36
Os07g0679700	B3 domain-containing protein Os07g0679700	-2.33
Os12g0591300	B3 domain-containing protein Os12g0591400	-2.03
OsI_36121	DNA replication licensing factor MCM2	1.50
Pabpc1	Polyadenylate-binding protein 1	10.61
PCMP-A5	Pentatricopeptide repeat-containing protein At3g26540	1.38
PCMP-E1	Pentatricopeptide repeat-containing protein At5g59600	1.28
PCMP-E4	Pentatricopeptide repeat-containing protein At1g03540	1.35
PCMP-E6	Pentatricopeptide repeat-containing protein At4g22760	-1.06
PCMP-E88	Pentatricopeptide repeat-containing protein At3g09040, mitochondrial	1.24
PCMP-E99	Pentatricopeptide repeat-containing protein At4g04370	1.64
PCMP-H12	Pentatricopeptide repeat-containing protein At1g08070, chloroplastic	1.15
PCMP-H53	Pentatricopeptide repeat-containing protein At4g33170	1.09
PCMP-H70	Pentatricopeptide repeat-containing protein At1g71420	1.58
PCMP-H81	Pentatricopeptide repeat-containing protein At3g57430, chloroplastic	1.15
PCNA	Proliferating cell nuclear antigen	1.35
PDCD4	Programmed cell death protein 4	8.93
PHN1	Protein argonaute PNH1	2.22
pif1	ATP-dependent DNA helicase pif1	-1.06
PIF1	ATP-dependent DNA helicase PIF1	-1.16
pif1	ATP-dependent DNA helicase pif1	2.99
pif1	ATP-dependent DNA helicase pif1	-1.14
PIF4	ATP-dependent DNA helicase PIF4	-1.03
PIF7	ATP-dependent DNA helicase PIF7	-1.15
PLA2	Protein terminal ear1 homolog	7.83
pol	Gag-Pol polyprotein	12.69
Pol	LINE-1 retrotransposable element ORF2 protein	-7.70
Ppie	Peptidyl-prolyl cis-trans isomerase E	1.51
R3hdm2	R3H domain-containing protein 2	4.37
RAD23D	Ubiquitin receptor RAD23d	4.11
RAD51	DNA repair protein RAD51 homolog	1.24
RAD5B	DNA repair protein RAD5B	1.49
RBG2	Glycine-rich RNA-binding protein 2, mitochondrial	3.55
RBG5	Glycine-rich RNA-binding protein 5, mitochondrial	3.17
RBG7	Glycine-rich RNA-binding protein GRP1A	3.14
RBP1	RNA-binding protein 1	4.60
RBP45A	Polyadenylate-binding protein RBP45A	2.64
RBP45C	Polyadenylate-binding protein RBP45C	4.12
RBP47B	Polyadenylate-binding protein RBP47B	4.58
RCOM_1506700	Probable aspartyl aminopeptidase	1.47
RECQSIM	ATP-dependent DNA helicase Q-like SIM	1.03
RH14	DEAD-box ATP-dependent RNA helicase 14	3.49
RH3	Histone H3.3	2.41
RH31	DEAD-box ATP-dependent RNA helicase 31	1.04
RNA1	RNA1 polyprotein	-14.32
RNA1	RNA1 polyprotein	-14.85
rnf2-a	E3 ubiquitin-protein ligase RING2-A	-1.04

rnhA	Ribonuclease HI	4.81
ROC3	Homeobox-leucine zipper protein ROC3	1.54
RPA1B	Replication protein A 70 kDa DNA-binding subunit B	1.58
RPT6A	26S proteasome regulatory subunit 8 homolog A	-2.34
RS2	Protein rough sheath 2	1.66
RS2Z33	Serine/arginine-rich splicing factor RS2Z33	2.60
RS40	Serine/arginine-rich splicing factor RS40	8.48
Rtfdc1	Replication termination factor 2	3.01
RZFP34	Probable E3 ubiquitin-protein ligase RZFP34	3.76
RZPF34	E3 ubiquitin-protein ligase RZFP34	2.72
S100a1	Protein S100-A1	11.53
SCL30A	Serine/arginine-rich SC35-like splicing factor SCL30A	3.82
SCL7	SCARECROW-LIKE protein 7	1.51
sde2	Replication stress response regulator SDE2	2.28
SKI2	DExH-box ATP-dependent RNA helicase DExH11	1.03
SKIP	SNW/SKI-interacting protein	8.67
SlH4	Histone H4	3.18
SlH4	Histone H4	2.26
Slx1b	Structure-specific endonuclease subunit SLX1	1.64
SNRNP40	U5 small nuclear ribonucleoprotein 40 kDa protein	8.69
snu13	NHP2-like protein 1	2.22
soH3-1	Histone H3.3b	-1.69
SPO11-1	Meiotic recombination protein SPO11-1	-3.15
SR45A	Serine/arginine-rich splicing factor SR45a	8.75
SRP72	Signal recognition particle subunit SRP72	-1.32
STI	Protein STICHEL	1.40
TE1	Protein terminal ear1	5.60
Tf2-12	Transposon Tf2-12 polyprotein	-8.51
Tnni2	Troponin I, fast skeletal muscle	-10.64
TOP2	DNA topoisomerase 2	2.33
TPR1	Protein TPR1	-1.39
TY2B-B	Transposon Ty2-B Gag-Pol polyprotein	2.43
TY3B-G	Transposon Ty3-G Gag-Pol polyprotein	6.38
TY3B-I	Transposon Ty3-I Gag-Pol polyprotein	9.48
TY4B-J	Transposon Ty4-J Gag-Pol polyprotein	-1.35
U2AF35B	Splicing factor U2af small subunit B	9.02
UBA1C	UBP1-associated proteins 1C	1.02
UBP1A	Oligouridylate-binding protein 1A	2.40
UVH3	DNA repair protein UVH3	-1.36
VIM	Vimentin	-9.63
Wrn	Werner syndrome ATP-dependent helicase homolog	2.07
XRCC2	DNA repair protein XRCC2 homolog	1.41
YBX1	Y-box-binding protein 1	11.39
ythdf2	YTH domain-containing family protein 2	3.60

Table S5. Cell morphogenesis genes differentially expressed in CR and CS.

Gene name	Protein name	$\log_2$ Ratio (CR/CS)
**Cell growth and development**		
3MMP	Metalloendoproteinase 3-MMP	-2.09
ACC1	Acetyl-CoA carboxylase 1	-1.47
ADF5	Actin-depolymerizing factor 5	2.11
AHL24	AT-hook motif nuclear-localized protein 24	-5.53
AIR3	Subtilisin-like protease SBT5.3	-5.84
AMP1	Probable glutamate carboxypeptidase AMP1	-1.30
ANXA2	Annexin A2	-8.83
AS2	ASYMMETRIC LEAVES 2	10.19
asp	Protein abnormal spindle	1.32
At1g06760	Histone H1	1.42
At1g51060	Probable histone H2A.1	1.90
At1g64390	Endoglucanase 6	-2.65
At1g67360	REF/SRPP-like protein At1g67360	9.30
At1g80180	MAPK kinase substrate protein At1g80180	-1.30
At2g32990	Endoglucanase 11	1.70
At3g01270	Probable pectate lyase 7	1.35
At3g05500	REF/SRPP-like protein At3g05500	4.84
At3g07010	Probable pectate lyase 8	5.25
At3g53190	Probable pectate lyase 12	1.80
At4g02290	Endoglucanase 17	5.02
At4g39010	Endoglucanase 24	-1.36
At5g13200	GEM-like protein 5	3.70
At5g27670	Probable histone H2A.5	2.59
At5g39980	Pentatricopeptide repeat-containing protein At5g39980, chloroplastic	1.10
At5g59910	Histone H2B.11	1.83
At5g45780	Probable LRR receptor-like serine/threonine-protein kinase At5g45780	1.49
At5g63180	Probable pectate lyase 22	-1.50
ATHB-13	Homeobox-leucine zipper protein ATHB-13	-1.31
AUR3	Serine/threonine-protein kinase Aurora-3	2.03
BPS1	Protein BPS1, chloroplastic	7.90
BTU1	Tubulin beta chain	3.37
BUBR1	Mitotic spindle checkpoint protein BUBR1	1.25
CAP1	Cyclase-associated protein 1	1.01
CEL1	Endoglucanase 8	3.29
CFL1	Cofilin-1	-10.22
Cfl2	Cofilin-2	10.07
CHR10	Probable helicase CHR10	-9.18
CHUP1	Protein CHUP1, chloroplastic	-4.05
CLASP	CLIP-associated protein	-1.43
CLPC1	Chaperone protein ClpC1, chloroplastic	3.04
COBL4	COBRA-like protein 4	1.25
COL1A1	Collagen alpha-1(I) chain	10.51
COL1A1	Collagen alpha-1(I) chain	-8.32
col1a2	Collagen alpha-2(I) chain	10.06
COL3A1	Collagen alpha-1(III) chain	-8.52

COL5A2	Collagen alpha-2(V) chain	-9.54
CR4	Serine/threonine-protein kinase-like protein CR4	2.31
CSLD4	Cellulose synthase-like protein D4	2.13
CSLD5	Cellulose synthase-like protein D5	1.56
CSLG3	Cellulose synthase-like protein G3	2.70
CSLH1	Cellulose synthase-like protein H1	-5.57
DCR	BAHD acyltransferase DCR	3.04
DGD2	Digalactosyldiacylglycerol synthase 2, chloroplastic	-1.82
DL	Protein DROOPING LEAF	5.58
DMP3	Protein DMP3	-2.21
DMP9	Protein DMP9	4.94
DRMH1	Dormancy-associated protein homolog 1	5.77
DRP5A	Dynamin-related protein 5A	1.42
DUF1	DUF724 domain-containing protein 1	1.15
DYAD	Protein DYAD	7.13
DYNLL1	Dynein light chain LC6, flagellar outer arm	1.40
EFEMP2	EGF-containing fibulin-like extracellular matrix protein 2	11.23
ERL2	LRR receptor-like serine/threonine-protein kinase ERL2	3.69
EXLB1	Expansin-like B1	8.86
EXPA11	Expansin-A11	3.74
EXPA4	Expansin-A4	1.70
EXPA6	Expansin-A6	-1.10
EXPA8	Expansin-A8	-2.05
EXT3	Extensin-3	-3.77
faeA	Feruloyl esterase A	-6.50
FEI1	LRR receptor-like serine/threonine-protein kinase FEI 1	1.39
FLA1	Fasciclin-like arabinogalactan protein 1	-1.22
FLNA	Filamin-A	-9.90
FLNB	Filamin-B	8.85
FN1	Fibronectin	-8.35
FTIP1	FT-interacting protein 1	1.27
GALS1	Galactan beta-1,4-galactosyltransferase GALS1	2.10
GATA15	GATA transcription factor 15	3.24
GATA18	GATA transcription factor 18	1.77
GLOX1	Aldehyde oxidase GLOX1	-2.41
GMGT1	Galactomannan galactosyltransferase 1	1.66
GPX4	Phospholipid hydroperoxide glutathione peroxidase	9.82
GSN	Gelsolin	9.00
Gsn	Gelsolin	-9.24
H2B.6	Histone H2B.6	3.33
HDG11	Homeobox-leucine zipper protein HDG11	2.53
HIS2B	Histone H2B	1.85
HMGB7	High mobility group B protein 7	1.53
HSL2	LRR receptor-like serine/threonine-protein kinase HSL2	2.46
IDD14	Protein indeterminate-domain 14	1.21
ISE2	DExH-box ATP-dependent RNA helicase DExH15 chloroplastic	-2.19
Itgb1	Integrin beta-1	-8.57
JGB	Protein JINGUBANG	5.43
KIN1	Kinesin-like protein KIN-1	3.51
KIN10A	Kinesin-like protein KIN-10A	1.59

KIN10B	Kinesin-like protein KIN-10B	2.26
KIN10C	Kinesin-like protein KIN-10C	1.17
KIN12D	Kinesin-like protein KIN-12D	1.35
KIN12E	Kinesin-like protein KIN-12E	1.74
KIN12F	Kinesin-like protein KIN-12F	1.21
KIN12F	Kinesin-like protein KIN-12F	2.68
KIN14D	Kinesin-like protein KIN-14D	1.03
KIN14K	Kinesin-like protein KIN-14K	-1.49
KIN14L	Kinesin-like protein KIN-14L	1.96
KIN14P	Kinesin-like protein KIN-14P	-1.66
KIN14R	Kinesin-like protein KIN-14R	1.24
KIN14S	Kinesin-like protein KIN-14S	1.77
KIN4A	Kinesin-like protein KIN-4A	1.02
KIN5D	Kinesin-like protein KIN-5D	1.35
KIN7D	Kinesin-like protein KIN-7D, mitochondrial	1.02
KIN7N	Kinesin-like protein KIN-7N	1.59
KIN8A	Kinesin-like protein KIN-8A	1.25
krt18	Keratin, type I cytoskeletal 18	10.63
LBD27	LOB domain-containing protein 27	1.71
LCV2	Protein LIKE COV 2	-1.35
LNG2	Protein LONGIFOLIA 2	3.64
LOX4	Lipoxygenase 4, chloroplastic	-3.99
MAIL1	Protein MAIN-LIKE 1	1.89
MAP65-3	65-kDa microtubule-associated protein 3	1.20
MIF2	Mini zinc finger protein 2	-8.44
MMD1	PHD finger protein MALE MEIOCYTE DEATH 1	-1.01
MMP2	72 kDa type IV collagenase	9.54
MMP2	72 kDa type IV collagenase	-6.40
MSP1	Leucine-rich repeat receptor protein kinase MSP1	4.24
Myl1	Myosin light chain 1/3, skeletal muscle isoform	-10.62
Myl6	Myosin light polypeptide 6	-11.06
Mylpf	Myosin regulatory light chain 2, skeletal muscle isoform	-11.03
NAC021	NAC domain-containing protein 21/22	2.26
NAC031	Protein CUP-SHAPED COTYLEDON 3	1.67
NAC056	NAC transcription factor 56	-1.71
NAC086	NAC domain-containing protein 86	1.40
NAC098	CUP-SHAPED COTYLEDON 2	3.58
NACK1	Kinesin-like protein NACK1	2.71
NACK2	Kinesin-like protein NACK2	1.77
NEK5	Serine/threonine-protein kinase Nek5	-1.61
NET2A	Protein NETWORKED 2A	-1.33
NET3C	Protein NETWORKED 3C	2.14
NPY1	BTB/POZ domain-containing protein NPY1	1.74
NUP133	Nuclear pore complex protein NUP133	1.04
OBE1	Protein OBERON 1	2.77
Os09g0533900	Endoglucanase 24	1.57
PAE11	Pectin acetylesterase 11	2.51
PAE7	Pectin acetylesterase 7	3.52
PAE8	Pectin acetylesterase 8	2.04
PALLD	Palladin	-8.78
PAP15	Purple acid phosphatase 15	-1.01

PCN	WD repeat-containing protein PCN	-1.24
PDF2	Homeobox-leucine zipper protein PROTODERMAL FACTOR 2	9.05
PHS1	Protein POOR HOMOLOGOUS SYNAPSIS 1	2.44
plaa2	Exopolygalacturonase	1.79
PME15	Probable pectinesterase 15	-8.82
PME17	Probable pectinesterase/pectinesterase inhibitor 17	3.08
PME32	Pectinesterase/pectinesterase inhibitor PPE8B	3.20
PME36	Probable pectinesterase/pectinesterase inhibitor 36	1.78
PME40	Probable pectinesterase/pectinesterase inhibitor 40	1.00
PME53	Probable pectinesterase 53	2.03
PME7	Probable pectinesterase/pectinesterase inhibitor 7	1.01
PMEI10	Pectinesterase inhibitor 10	2.80
PPME1	Pectinesterase PPME1	-1.66
PPME1	Pectinesterase 2	1.56
PS1	FHA domain-containing protein PS1	2.31
PTD	PARTING DANCERS	2.20
PTL	Trihelix transcription factor PTL	1.11
PXC1	Leucine-rich repeat receptor-like protein kinase PXC1	1.36
QKY	QUIRKY	1.77
QUA2	Probable pectin methyltransferase QUA2	1.37
REF6	Lysine-specific demethylase REF6	-3.17
RHD3	Protein ROOT HAIR DEFECTIVE 3	2.87
RHF2A	E3 ubiquitin-protein ligase RHF2A	4.57
RIC4	CRIB domain-containing protein RIC4	-1.15
RMD1	Sporulation protein RMD1	1.12
RPD1	Protein ROOT PRIMORDIUM DEFECTIVE 1	-1.12
RPN12A	26S proteasome non-ATPase regulatory subunit 8 homolog A	2.46
RTNLB1	Reticulon-like protein B1	3.82
SERPINH1	Serpin H1	9.51
SG1	protein SLOW GREEN 1, chloroplastic	-1.01
SIA1	Sialyltransferase-like protein 1	1.01
Smg9	Protein SMG9	1.25
SP1L1	SPIRAL1-like 1	1.98
SRO1	Probable inactive poly [ADP-ribose] polymerase SRO1	4.00
TKPR1	Tetraketide alpha-pyrone reductase 1	3.66
TKPR2	Tetraketide alpha-pyrone reductase 2	1.30
TMSB10	Thymosin beta-10	-12.70
Tmsb4x	Thymosin beta-4	-11.35
Tmsb4x	Thymosin beta-11	12.19
TOR1L2	TORTIFOLIA1-like protein 2	2.59
TOR1L4	TORTIFOLIA1-like protein 4	1.06
TPD1	Protein TAPETUM DETERMINANT 1	9.90
TPM4	Tropomyosin alpha-4 chain	-8.67
TUBA	Tubulin alpha chain	2.64
tuba	Tubulin alpha chain	10.27
TUBA	Tubulin alpha chain	3.09
TUBA	Tubulin alpha-2 chain	-1.02
TUBA1A	Tubulin alpha-1A chain	9.13
TUBA3	Tubulin alpha-3 chain	-1.09
TUBA6	Tubulin alpha-6 chain	2.47

TUBB	Tubulin beta chain	-9.30
TUBB1	Tubulin beta-1 chain	2.29
tubb4	Tubulin beta chain	1.68
TUBB4	Tubulin beta-4 chain	2.05
TUBB4B	Tubulin beta-3 chain	-8.77
TUBB4B	Tubulin beta-4B chain	5.62
ULT2	Protein ULTRAPETALA 2	1.60
VQ29	VQ motif-containing protein 29	9.25
WAV3	E3 ubiquitin-protein ligase WAV3	4.27
Wdr13	WD repeat-containing protein 13	-1.12
WDR55	WD repeat-containing protein 55	1.87
WLIM2B	LIM domain-containing protein WLIM2b	2.72
WOX1	WUSCHEL-related homeobox 1	4.16
WOX11	WUSCHEL-related homeobox 11	9.30
WOX4	WUSCHEL-related homeobox 4	1.75
WOX8	WUSCHEL-related homeobox 8	-1.07
WOX9	WUSCHEL-related homeobox 9	5.79
WRAP73	WD repeat-containing protein WRAP73	1.00
XTH8	Probable xyloglucan endotransglucosylase/hydrolase protein 8	3.28
YAB1	Axial regulator YABBY 1	15.61
YAB5	Axial regulator YABBY 5	9.27
ZPR1	LITTLE ZIPPER 1	-1.03
**Cell division/cell differentiation**		
5-Aug	AUGMIN subunit 5	-1.52
ACL5	Thermospermine synthase ACAULIS5	2.06
At3g09070	Protein OCTOPUS	1.09
At3g51280	Protein POLLENLESS 3-LIKE 2	3.42
AUR1	Serine/threonine-protein kinase Aurora-1	2.18
BAM1	Leucine-rich repeat receptor-like serine/threonine-protein kinase BAM1	1.59
CCNG1	Cyclin-G1	11.37
CDC2	Cell division control protein 2 homolog	1.02
CDC20-1	Cell division cycle 20.1, cofactor of APC complex	3.07
CDC25	Dual specificity phosphatase Cdc25	1.02
ATHB-14	Homeobox-leucine zipper protein ATHB-14	1.33
CYCB2-3	G2/mitotic-specific cyclin-2	2.86
CYCB2-4	G2/mitotic-specific cyclin-1	2.44
CYCD4-2	Cyclin-D4-2	1.21
DIM1A	Ribosomal RNA small subunit methyltransferase	-2.70
EB1C	Microtubule-associated protein RP/EB family member 1C	1.11
EDE1	Protein ENDOSPERM DEFECTIVE 1	2.78
EPFL2	EPIDERMAL PATTERNING FACTOR-like protein 2	1.54
EPFL4	EPIDERMAL PATTERNING FACTOR-like protein 4	2.39
ERF11	Ethylene-responsive transcription factor 11	9.54
FRL4A	FRIGIDA-like protein 4a	2.80
Fstl1	Follistatin-related protein 1	8.89
FZR3	Protein FIZZY-RELATED 3	2.07
GIF1	GRF1-interacting factor 1	2.57
HD3A	Protein HEADING DATE 3A	5.11
MAD2	Mitotic spindle checkpoint protein MAD2	1.06
MAP65-1	65-kDa microtubule-associated protein 1	1.67

MS5	Protein POLLENLESS 3	2.62
ncapd2	Condensin complex subunit 1	1.91
ncapg	Condensin complex subunit 3	1.15
PATL1	Patellin-1	3.60
PATL3	Patellin-3	-1.07
PATL6	Patellin-6	1.40
REV	Homeobox-leucine zipper protein REVOLUTA	1.46
SCIN	Adseverin	8.78
SCL6	Scarecrow-like protein 6	1.25
SPC25	Kinetochore protein SPC25 homolog	1.07
SPL8	Squamosa promoter-binding-like protein 8	6.55
SYN1	Sister chromatid cohesion 1 protein 1	4.49
SYN2	Sister chromatid cohesion 1 protein 2	1.27
TAGLN	Transgelin	-10.97
TAGLN	Transgelin	8.83
TPX2	Protein TPX2	2.65
UFO	Protein UNUSUAL FLORAL ORGANS	11.58
WUS	Protein WUSCHEL	4.95
**Nutrient reservoir**		
At2g18540	Vicilin-like seed storage protein At2g18540	1.47
AT2S1	2S seed storage protein 1	-5.29
AT2S2	2S seed storage protein 2	-4.29
AT2S3	2S seed storage protein 3	-9.95
At4g25140	Oleosin 18.5 kDa	10.44
CRA1	12S seed storage protein CRA1	-4.77
CRB	12S seed storage protein CRB	-3.86
CRC	12S seed storage protein CRC	-3.42
CRD	12S seed storage protein CRD	1.56
GLUA1	Glutelin type-A 1	3.44
LEGA	11S globulin seed storage protein 2	8.31
LEGB	Legumin B	10.17
MATP6-A	Oleosin 18.2 kDa	-1.44
**Cell redox homeostasis**		
At1g08570	Thioredoxin-like 1-1, chloroplastic	1.20
At2g35010	Thioredoxin O1, mitochondrial	1.01
CNGC18	Cyclic nucleotide-gated ion channel 18	2.57
PDIA3	Protein disulfide-isomerase A3	-9.19
PDIL2-1	disulfide-isomerase like 2-1	2.07
TIL	Temperature-induced lipocalin-1	3.60
**Others**		
N/A	Early nodulin-93	1.91
N/A	Macrophage migration inhibitory factor homolog	1.40
ASP	21 kDa seed protein	2.21
ASY1	Meiosis-specific protein ASY1	1.61
BRPF3	Bromodomain and PHD finger-containing protein 3	2.37
CALD1	Caldesmon	-9.92
FAN1	Fanconi-associated nuclease 1 homolog	-1.31

Table S6. Bio-signaling genes differentially expressed in CR and CS.

Gene name	Protein name	log_2 Ratio (CR/CS)
**Signal transduction**		
N/A	Remorin	4.67
Ankrd13c	Ankyrin repeat domain-containing protein 13C	2.01
ANNAT8	Annexin-like protein RJ4	-2.61
ANXA1	Annexin A1	10.06
ANXA2	Annexin A2	-9.84
anxa2-a	Annexin A2-A	11.36
ANXA5	Annexin A5	-8.90
At3g19850	BTB/POZ domain-containing protein At3g19850	-9.17
At4g30940	BTB/POZ domain-containing protein At4g30940	2.24
ATL29	RING-H2 finger protein ATL29	2.06
ATL67	RING-H2 finger protein ATL67	4.60
BAG1	BAG family molecular chaperone regulator 1	2.00
BEH1	BES1/BZR1 homolog protein 1	9.73
BETVIII	Calcium-binding allergen Bet v 3	-1.05
BI-1	Bax inhibitor 1	4.06
BKI1	BRI1 kinase inhibitor 1	2.32
C/VIF1	Cell wall / vacuolar inhibitor of fructosidase 1	2.97
CAM3	Calmodulin-3	8.07
CBL7	Calcineurin B-like protein 7	2.20
CCM1	Calmodulin	9.18
CCT2	T-complex protein 1 subunit beta	8.74
CD63	CD63 antigen	-9.30
CET2	CEN-like protein 2	-7.60
Cfd	Complement factor D	-9.99
CIPK10	CBL-interacting serine/threonine-protein kinase 10	-1.51
CIPK13	CBL-interacting serine/threonine-protein kinase 13	8.21
CIPK25	CBL-interacting serine/threonine-protein kinase 25	3.18
CIPK3	CBL-interacting serine/threonine-protein kinase 3	-1.00
CIPK5	CBL-interacting protein kinase 5	-1.05
CIPK5	CBL-interacting serine/threonine-protein kinase 5	3.20
CLE27	CLAVATA3/ESR (CLE)-related protein 27	1.03
CLE3	CLAVATA3/ESR (CLE)-related protein 3	2.67
CLU	Clusterin	-9.31
CML14	Probable calcium-binding protein CML14	1.89
CML18	Probable calcium-binding protein CML18	1.34
CML18	Probable calcium-binding protein CML18	8.48
CML28	Probable calcium-binding protein CML28	-1.41
CML41	Probable calcium-binding protein CML41	2.41
CML42	Calcium-binding protein CML42	4.58
CML46	Probable calcium-binding protein CML46	-3.75
COL1A2	Collagen alpha-2(I) chain	-7.09
Col1a2	Collagen alpha-2(I) chain	-9.84
CP1	Calcium-binding protein CP1	2.56
CPR1	F-box protein CPR1	-8.47
crabp2	Cellular retinoic acid-binding protein 2	9.84
DCN	Decorin	-7.60

ERDJ3B	DnaJ protein ERDJ3B	3.54
FD	Protein FD	-1.58
Flna	Filamin-A	9.77
Fth1	Ferritin heavy chain	-10.63
GAD4	Glutamate decarboxylase 4	3.43
GDU1	Protein GLUTAMINE DUMPER 1	4.09
GG3	Guanine nucleotide-binding protein subunit gamma 3	-2.21
GNAS	Guanine nucleotide-binding protein G(s) subunit alpha isoforms XLas	-8.56
gnb2l1	Guanine nucleotide-binding protein subunit beta-2-like 1	12.10
GNL2	ARF guanine-nucleotide exchange factor GNL2	1.14
GPI8	GPI-anchor transamidase	1.10
GRF6	14-3-3-like protein GF14 lambda	2.26
Gtp-bp	Signal recognition particle receptor subunit alpha homolog	-1.14
H2-D1	H-2 class I histocompatibility antigen, D-B alpha chain	-9.68
HMGA	HMG-Y-related protein A	2.14
HSPA8	Heat shock cognate 71 kDa protein	-8.90
HTH	Protein HOTHEAD	-7.81
HTRA1	Serine protease HTRA1	-10.05
htra1a	Serine protease HTRA1A	8.90
IFITM1	Interferon-induced transmembrane protein 1	9.90
IP5P7	Type IV inositol polyphosphate 5-phosphatase 7	1.37
IQM1	IQ domain-containing protein IQM1	-1.98
Itm2b	Integral membrane protein 2B	-9.51
KRT8	Keratin, type II cytoskeletal 8	-5.95
LFG2	Protein LIFEGUARD 2	2.16
MAIL3	Serine/threonine-protein phosphatase 7 long form homolog	-12.06
MAKR1	Probable membrane-associated kinase regulator 1	1.65
MYL9	Myosin regulatory light polypeptide 9	-9.88
NAC048	NAC domain-containing protein 48	-4.07
NBP35	Cytosolic Fe-S cluster assembly factor NBP35	3.50
Nupr1	Nuclear protein 1	11.10
Otud3	OTU domain-containing protein 3	2.82
PAT07	Probable protein S-acyltransferase 7	1.40
PBLD	Phenazine biosynthesis-like domain-containing protein	4.10
PDIL1-2	Protein disulfide isomerase-like 1-2	2.20
PFN1	Profilin-1	-10.29
PGIP1	Polygalacturonase inhibitor 1	3.74
PHR2	Blue-light photoreceptor PHR2	-1.14
Psap	Prosaposin	-9.33
PSAP	Prosaposin	-9.13
RAB11B	Ras-related protein Rab11B	2.51
RALF	Rapid alkalinization factor	-1.08
RALFL4	Protein RALF-like 4	-1.75
RCOM_0864260	CASP-like protein 2B1	2.23
Rlc-a	Myosin regulatory light chain 2, smooth muscle minor isoform	-9.82
ROPGAP2	Rho GTPase-activating protein 2	1.28
ROPGEF1	Rop guanine nucleotide exchange factor 1	1.85
ROPGEF5	Rop guanine nucleotide exchange factor 5	1.88
RPS3	40S ribosomal protein S3	-8.95
S100A14	Protein S100-A14	9.02

S100A16	Protein S100-A16	9.62
S100B	Protein S100-B	9.66
Scgb1a1	Uteroglobin	-11.73
SDI1	Protein SULFUR DEFICIENCY-INDUCED 1	-1.24
SEC11A	Signal peptidase complex catalytic subunit SEC11A	4.07
Serpinb1b	Leukocyte elastase inhibitor B	9.67
SERPINE1	Plasminogen activator inhibitor 1	-9.07
SLC25A6	ADP/ATP translocase 3	-7.74
SPARC	SPARC	-8.62
Srprb	Signal recognition particle receptor subunit beta	1.11
SRR1	Protein SENSITIVITY TO RED LIGHT REDUCED 1	-1.25
Stxbp5	Syntaxin-binding protein 5	-1.21
TGM2	Protein-glutamine gamma-glutamyltransferase 2	-9.91
Tnfrsf11b	Tumor necrosis factor receptor superfamily member 11B	9.94
TNNC2	Troponin C, skeletal muscle	-11.21
Tnnt3	Troponin T, fast skeletal muscle	-11.06
**Hormone mediated signaling pathway**		
N/A	Auxin-induced protein X10A	-4.94
ABI1	Protein phosphatase 2C 56	1.45
AOC	Allene oxide cyclase, chloroplastic	-3.20
ARF19	Auxin response factor 19	1.04
ARF5	Auxin response factor 5	2.24
ARF9	Auxin response factor 9	2.52
ARR17	Two-component response regulator ARR17	3.74
ARR9	Two-component response regulator ARR9	-1.59
At1g54200	Protein BIG GRAIN 1-like B	2.27
At1g80440	F-box/kelch-repeat protein At1g80440	4.47
At2g30020	Probable protein phosphatase 2C 25	-1.10
At4g13040	Ethylene-responsive transcription factor-like protein At4g13040	-1.57
AUX22D	Auxin-induced protein 22D	2.53
CDKB2-2	Cyclin-dependent kinase B2-2	2.08
CIP1	COP1-interactive protein 1	-1.05
CUL1	Cullin-1	-9.35
DRM1	Auxin-repressed 12.5 kDa protein	-1.44
EBF2	EIN3-binding F-box protein 2	-1.23
EIN3	ETHYLENE INSENSITIVE 3	-1.14
ERF010	Ethylene-responsive transcription factor ERF010	-2.43
ERF034	Ethylene-responsive transcription factor ERF034	2.05
ERF061	Ethylene-responsive transcription factor ERF061	-1.60
ERF071	Ethylene-responsive transcription factor ERF071	2.69
ERF073	Ethylene-responsive transcription factor ERF073	2.07
ERF087	Ethylene-responsive transcription factor ERF087	-2.69
ERF110	Ethylene-responsive transcription factor ERF110	-5.90
ERF14	Ethylene-responsive transcription factor 14	8.78
ERF1B	Ethylene-responsive transcription factor 1B	-4.65
ERF2	Ethylene-responsive transcription factor 2	9.52
ERF5	Ethylene-responsive transcription factor 5	-3.21
GAIPB	DELLA protein GAIP-B	2.35
GASA11	Gibberellin-regulated protein 11	-6.63
GASA4	Gibberellin-regulated protein 4	8.57
HAB1	Protein phosphatase 2C 16	1.61

HLS1	Probable N-acetyltransferase HLS1	-3.34
IAA1	Auxin-responsive protein IAA1	2.16
IAA13	Auxin-responsive protein IAA13	1.16
IAA20	Auxin-responsive protein IAA20	5.53
IAA26	Auxin-responsive protein IAA26	1.41
IAA31	Auxin-responsive protein IAA31	10.00
IAA8	Auxin-responsive protein IAA8	1.76
IAA9	Auxin-responsive protein IAA9	3.21
KEG	E3 ubiquitin-protein ligase KEG	-10.13
LRP1	Protein LATERAL ROOT PRIMORDIUM 1	2.24
MARD1	Protein MARD1	1.89
MSPR10-1	Major allergen Pru ar 1	-2.57
NHL6	NDR1/HIN1-like protein 6	-2.94
NPR6	Regulatory protein NPR6	1.13
PARC	Probable glutathione S-transferase	-2.38
PCKR3	Major allergen Api g 1, isoallergen 2	4.66
PIN1B	Probable auxin efflux carrier component 1b	1.99
PIN1C	Probable auxin efflux carrier component 1c	2.61
PIN6	Auxin efflux carrier component 6	-1.96
PRP1	Probable glutathione S-transferase	-1.76
PYL5	Abscisic acid receptor PYL5	2.41
PYL7	Abscisic acid receptor PYL7	3.67
PYR1	Abscisic acid receptor PYR1	3.14
RAP2-3	Ethylene-responsive transcription factor RAP2-3	-2.68
RGLG1	E3 ubiquitin-protein ligase RGLG1	3.98
RNL	tRNA ligase 1	1.94
RR10	Two-component response regulator ORR10	-1.46
SAPK2	Serine/threonine-protein kinase SAPK2	1.13
SAUR32	Auxin-responsive protein SAUR32	-1.92
SAUR36	Auxin-responsive protein SAUR36	-2.51
SAUR50	Auxin-responsive protein SAUR50	4.58
SAUR71	Auxin-responsive protein SAUR71	-2.95
SAUR72	Auxin-responsive protein SAUR72	-1.65
SHN3	Ethylene-responsive transcription factor SHINE 3	-2.03
SKP1A	SKP1-like protein 1A	8.70
SRK2E	Serine/threonine-protein kinase SRK2E	3.47
SRT1	NAD-dependent protein deacetylase SRT1	1.01
SRT2	NAD-dependent protein deacylase SRT2	1.00
TIFY6B	Protein TIFY 6B	-1.08
TIMP1	Metalloproteinase inhibitor 1	-10.48
Timp2	Metalloproteinase inhibitor 2	11.30
TPL	Protein TOPLESS	-1.44
TTL1	TPR repeat-containing thioredoxin TTL1	1.22
VAB	VAN3-binding protein	2.04
**Protein kinase**		
ACTA2	Actin, aortic smooth muscle	-8.28
AFC2	Serine/threonine-protein kinase AFC2	-1.64
ASK3	Shaggy-related protein kinase gamma	3.30
At1g12460	Probable LRR receptor-like serine/threonine-protein kinase At1g12460	2.01
At2g23950	Probable LRR receptor-like serine/threonine-protein kinase At2g23950	1.64
At2g24230	Probable LRR receptor-like serine/threonine-protein kinase At2g24230	1.70

At2g40270	Inactive receptor-like serine/threonine-protein kinase At2g40270	8.42
At2g44130	F-box/kelch-repeat protein At2g44130	-1.21
At3g03770	Probable inactive leucine-rich repeat receptor-like protein kinase At3g03770	1.91
At3g05640	Probable protein phosphatase 2C 34	-3.02
At3g47570	Probable LRR receptor-like serine/threonine-protein kinase At3g47570	-1.02
At3g51470	Probable protein phosphatase 2C 47	4.39
At3g51990	Serine/threonine-protein kinase-like protein At3g51990	-1.02
At4g28400	Probable protein phosphatase 2C 58	-1.37
At4g36180	Probable LRR receptor-like serine/threonine-protein kinase At4g36180	1.77
At5g05200	Uncharacterized aarF domain-containing protein kinase At5g05200, chloroplastic	-1.03
At5g18400	Anamorsin homolog	3.29
At5g67200	Probable inactive receptor kinase At5g67200	1.26
ATPK2	Serine/threonine-protein kinase AtPK2/AtPK19	8.79
ATR	Serine/threonine-protein kinase ATR	1.90
BLUS1	Serine/threonine-protein kinase BLUS1	-1.96
BRL2	Brassinosteroid LRR receptor kinase BRL2	-4.01
BSK2	Serine/threonine-protein kinase BSK2	1.64
BSL1	Serine/threonine-protein phosphatase BSL1	4.89
CCR2	Serine/threonine-protein kinase-like protein CCR2	2.06
Cdc7	Cell division cycle 7-related protein kinase	1.41
Ckm	Creatine kinase M-type	-11.11
CRK25	Cysteine-rich receptor-like protein kinase 25	-1.99
CRK36	Cysteine-rich receptor-like protein kinase 36	1.01
CYCA1-1	Cyclin-A1-1	3.45
CYCA2-1	Cyclin-A2-1	1.39
CYCA2-4	Cyclin-A2-4	1.90
CYCA3-2	Cyclin-A3-2	2.06
CYCD3-3	Cyclin-D3-3	1.12
CYCD4-1	Cyclin-D4-1	1.07
CYCD5-1	Cyclin-D5-1	1.05
CYCD6-1	Cyclin-D6-1	-1.77
CYCP3-1	Cyclin-P3-1	2.03
CYCU4-1	Cyclin-U4-1	-1.49
D6PKL2	Protein kinase PVPK-1	1.00
GRIK2	Serine/threonine-protein kinase GRIK2	-1.18
HD16	Casein kinase 1-like protein HD16	4.63
HT1	Serine/threonine/tyrosine-protein kinase HT1	3.42
ILK1	Integrin-linked protein kinase 1	2.28
IMK3	Probable leucine-rich repeat receptor-like protein kinase IMK3	3.72
KINB2	SNF1-related protein kinase regulatory subunit beta-2	-2.13
KING1	SNF1-related protein kinase regulatory subunit gamma-1	9.17
KRP7	Cyclin-dependent kinase inhibitor 7	1.73
LECRKS5	Probable L-type lectin-domain containing receptor kinase S.5	-1.63
LYK2	Protein LYK2	3.27
LYK5	LYK5	-2.71
MAPKKK17	Mitogen-activated protein kinase kinase kinase 17	-1.22
MIK1	MDIS1-interacting receptor like kinase 1	5.61
MKK9	Mitogen-activated protein kinase kinase 9	-2.16
MPK3	Mitogen-activated protein kinase 3	-2.71

NEK2	Serine/threonine-protein kinase Nek2	1.10
NEK6	Serine/threonine-protein kinase Nek6	-1.46
NIK3	Protein NSP-INTERACTING KINASE 3	2.51
NPG1	Protein NPG1	1.41
Os01g0656200	Probable protein phosphatase 2C 8	-2.84
OsI_35105	Pyruvate kinase 1, cytosolic	3.51
OXI1	Serine/threonine-protein kinase OXI1	9.39
PBL10	Probable serine/threonine-protein kinase PBL10	-1.20
PERK1	Proline-rich receptor-like protein kinase PERK1	1.80
PERK10	Proline-rich receptor-like protein kinase PERK10	1.39
PERK3	Proline-rich receptor-like protein kinase PERK3	1.81
PERK4	Proline-rich receptor-like protein kinase PERK4	-3.16
PHY1	Light-sensor Protein kinase	1.59
PID	Protein kinase PINOID	3.87
PIP5K2	Phosphatidylinositol 4-phosphate 5-kinase 2	1.02
PPCK2	Phosphoenolpyruvate carboxylase kinase 2	8.99
PPDK	Pyruvate, phosphate dikinase 1, chloroplastic	4.08
PPX2	Serine/threonine-protein phosphatase PP-X isozyme 2	3.52
PRK4	Pollen receptor-like kinase 4	-1.65
PSKR1	Phytosulfokine receptor 1	-4.69
PSY1R	Tyrosine-sulfated glycopeptide receptor 1	-3.74
PTI13	PTI1-like tyrosine-protein kinase 3	5.00
PUB14	U-box domain-containing protein 14	3.31
PUB34	U-box domain-containing protein 34	-1.21
PUB38	U-box domain-containing protein 38	-1.50
PUB51	U-box domain-containing protein 51	-1.80
PV42A	SNF1-related protein kinase regulatory subunit gamma-like PV42a	-3.56
PXC3	Leucine-rich repeat receptor-like tyrosine-protein kinase PXC3	2.57
RCH2	Receptor-like protein kinase 2	-1.17
RGI3	LRR receptor-like serine/threonine-protein kinase	1.60
RKL1	Probable inactive receptor kinase At1g48480	1.61
SMR15	Cyclin-dependent protein kinase inhibitor SMR15	1.12
THE1	Receptor-like protein kinase THESEUS 1	-1.15
TIO	Serine/threonine-protein kinase TIO	-2.39
TMK1	Receptor protein kinase TMK1	-1.18
V-FGR	Tyrosine-protein kinase transforming protein Fgr	12.40
WAKL15	Wall-associated receptor kinase-like 15	3.06
WAKL20	Wall-associated receptor kinase-like 20	-4.77
wif1	Wnt inhibitory factor 1	10.11
WNK1	Serine/threonine-protein kinase WNK1	1.57
WNK11	Probable serine/threonine-protein kinase WNK11	1.39
XLG3	Extra-large guanine nucleotide-binding protein 3	-1.98

Table S7. Stress response genes differentially expressed in CR and CS.

Gene name	Protein name	log_2 Ratio (CR/CS)
**Heat shock protein**		
AHSA1	Activator of 90 kDa heat shock protein ATPase homolog 1	3.29
hsc71	Heat shock cognate 70 kDa protein	-8.44
HSP17.6	17.6 kDa class II heat shock protein	6.06
HSP17.6B	17.6 kDa class I heat shock protein 2	8.02
HSP17.6C	17.6 kDa class I heat shock protein 3	4.72
HSP17.7	17.1 kDa class II heat shock protein	1.44
HSP18.1	18.1 kDa class I heat shock protein	9.64
HSP18.2	18.2 kDa class I heat shock protein	3.72
HSP18.5-C	18.5 kDa class I heat shock protein	1.01
HSP22.0	22.0 kDa class IV heat shock protein	2.51
HSP23.6	23.6 kDa heat shock protein, mitochondrial	-4.44
HSP26.5	26.5 kDa heat shock protein, mitochondrial	8.75
HSP70-14	Heat shock 70 kDa protein 14	4.41
HSP70-5	Heat shock 70 kDa protein 5	9.70
HSP70-9	Heat shock 70 kDa protein 9, mitochondrial	8.64
HSP90-2	Heat shock protein 90-2	3.13
HSP90-4	Heat shock protein 90-4	8.55
HSP90AA1	Heat shock protein HSP 90-alpha	-9.07
HSPA8	Heat shock cognate 71 kDa protein	-8.90
hspa8	Heat shock cognate 71 kDa protein	8.20
HSP70	Heat shock cognate 70 kDa protein	-1.11
**Abiotic stress**		
N/A	Desiccation-related protein PCC13-62	1.34
ABCG11	ABC transporter G family member 11	7.35
ACR8	ACT domain-containing protein ACR8	2.54
ACT2	Actin-2	1.78
ADH1	Alcohol dehydrogenase class-P	3.79
AHK4	Histidine kinase 4	1.75
ALDH3H1	Aldehyde dehydrogenase family 3 member H1	-2.47
APS2	ATP sulfurylase 2	-1.60
ARG7	Indole-3-acetic acid-induced protein ARG7	1.32
ARIA	ARM REPEAT PROTEIN INTERACTING WITH ABF2	1.28
At1g04770	Protein SULFUR DEFICIENCY-INDUCED 2	3.32
At1g18250	Osmotin-like protein	1.55
At1g59620	Probable disease resistance protein At1g59620	-1.70
At1g75040	Pathogenesis-related protein 5	-6.12
At2g14610	Pathogenesis-related protein 1	-8.99
At3g16530	Lectin-like protein At3g16530	3.64
At3g17800	UV-B-induced protein At3g17800, chloroplastic	8.41
At3g26430	Esterase	-2.62
At3g28600	AAA-ATPase At3g28600	-10.18
At4g32940	Vacuolar-processing enzyme gamma-isozyme	3.22
At5g05600	Probable 2-oxoglutarate-dependent dioxygenase At5g05600	-4.10
ATJ8	Chaperone protein dnaJ 8, chloroplastic	3.29
ATL24	NEP1-interacting protein-like 2	3.73
BAP1	BON1-associated protein 1	3.46

BIP4	Luminal-binding protein 4	-1.68
BSMT1	Salicylate/benzoate carboxyl methyltransferase	-1.13
BZIP9	Basic leucine zipper 9	1.97
CAMBP25	Calmodulin-binding protein 25	1.57
CAMTA5	Calmodulin-binding transcription activator 5	-2.48
CAT1	Catalase-1	3.95
CAT2	Catalase-2	4.88
CAT3	Catalase-3	2.98
CHY1	3-hydroxyisobutyryl-CoA hydrolase 1	1.24
CIPK21	CBL-interacting serine/threonine-protein kinase 21	-2.57
CML9	Calmodulin-like protein 9	2.62
Col3a1	Collagen alpha-1(III) chain	-10.35
COPT6	Copper transporter 6	3.78
CP12-3	Calvin cycle protein CP12-3, chloroplastic	4.45
CPC	Transcription factor CPC	-1.10
CRPK1	Cold-responsive protein kinase 1	-1.04
CRRSP55	Cysteine-rich repeat secretory protein 55	3.58
CRT1	Calreticulin-1	2.10
CYCLASE1	Cyclase-like protein 1	3.76
CYP81F2	Cytochrome P450 81F2	3.43
CYS4	Cysteine proteinase inhibitor 4	9.69
CYS6	Cysteine proteinase inhibitor 6	4.95
DRM1	Dormancy-associated protein 1	4.72
ERD14	Dehydrin ERD14	4.04
ERD15	Protein EARLY RESPONSIVE TO DEHYDRATION 15	3.80
ERD7	Protein EARLY-RESPONSIVE TO DEHYDRATION 7, chloroplastic	4.27
ERECTA	LRR receptor-like serine/threonine-protein kinase ERECTA	3.69
ERF012	Ethylene-responsive transcription factor ERF012	4.50
EXL2	Protein EXORDIUM-like 2	3.50
FAD-OXR	Berberine bridge enzyme-like 22	3.06
FAMT	Farnesoic acid carboxyl-O-methyltransferase	-13.41
FAX5	Protein FATTY ACID EXPORT 5	8.38
FQR1	NAD(P)H dehydrogenase (quinone) FQR1	1.86
GH3.1	Probable indole-3-acetic acid-amido synthetase GH3.1	5.84
GH3.6	Probable indole-3-acetic acid-amido synthetase GH3.6	-1.42
HAT22	Homeobox-leucine zipper protein HAT22	2.07
Hba	Hemoglobin subunit alpha	-11.54
Hbb-b1	Hemoglobin subunit beta-1	-11.83
HDT3	Histone deacetylase HDT3	2.97
HSPRO2	Nematode resistance protein-like HSPRO2	9.01
HVA22A	HVA22-like protein a	-1.32
krt8	Intermediate filament protein ON3	9.66
LA1	Protein LAZY 1	1.64
LECRK2	G-type lectin S-receptor-like serine/threonine-protein kinase LECRK2	-4.86
LECRK3	G-type lectin S-receptor-like serine/threonine-protein kinase LECRK3	4.03
LRR1	Leucine-rich repeat protein 1	2.30
LTI65	Low-temperature-induced 65 kDa protein	-1.06
MED37D	Probable mediator of RNA polymerase II transcription subunit 37c	2.43
MED37F	Mediator of RNA polymerase II transcription subunit 37f	2.03
MEE14	CCG-binding protein 1	3.91
MGL	Methionine gamma-lyase	11.82

MLP329	MLP-like protein 329	-1.22
MLP34	MLP-like protein 34	-8.09
Mmp2	72 kDa type IV collagenase	9.70
MPSR1	E3 ubiquitin-protein ligase MPSR1	4.48
Mtnd1	NADH-ubiquinone oxidoreductase chain 1	-10.76
MYB102	Transcription factor MYB102	-4.17
MYB16	Transcription factor MYB16	2.77
MYB2	Transcription factor MYB2	-5.60
MYB20	Transcription factor MYB20	2.02
MYB3	Transcription factor MYB3	-2.45
MYB5	Transcription repressor MYB5	-1.42
MYB78	Transcription factor MYB78	-1.90
NAC002	NAC domain-containing protein 2	3.78
NAC102	NAC domain-containing protein 102	3.03
NDA1	Internal alternative NAD(P)H-ubiquinone oxidoreductase A1, mitochondrial	-1.40
NFD4	NUCLEAR FUSION DEFECTIVE 4	2.65
NOI4	Protein NOI4	4.02
NPF4.3	Protein NRT1/ PTR FAMILY 4.3	-3.18
NPR1	BTB/POZ domain and ankyrin repeat-containing protein NPR1	-1.23
ORS1	Protein ORGAN SIZE RELATED 1	3.08
PBL16	Probable serine/threonine-protein kinase PBL16	1.15
PBL23	Probable serine/threonine-protein kinase PBL23	1.28
PBP1	Calcium-binding protein PBP1	2.79
PCAP1	Plasma membrane-associated cation-binding protein 1	3.26
PCO1	Plant cysteine oxidase 1	3.27
PCO2	Plant cysteine oxidase 2	3.42
PCR2	Protein PLANT CADMIUM RESISTANCE 2	9.46
PDC2	Pyruvate decarboxylase 2	6.48
PDIL2-3	disulfide-isomerase 2-3	3.59
PER19	Peroxidase 19	-1.76
PER26	Probable peroxidase 26	2.47
PER40	Peroxidase 40	6.73
PI4KG5	Phosphatidylinositol 4-kinase gamma 5	3.19
PICBP	Calmodulin binding protein PICBP	-2.36
PLAT1	PLAT domain-containing protein 1	3.96
PLDALPHA 4	Phospholipase D alpha 4	1.13
PRB1	Pathogenesis-related protein 1	-3.91
PRUA1	Major allergen Pru av 1	-1.22
PS2	Inorganic pyrophosphatase 1	4.40
PUB21	U-box domain-containing protein 21	-3.33
PUB26	U-box domain-containing protein 26	2.83
PYM	POLYCHOME	1.35
RACK1	Receptor of activated protein C kinase 1	-8.43
RBG4	Glycine-rich RNA-binding protein 4, mitochondrial	1.54
RBOHD	Respiratory burst oxidase homolog protein D	3.01
RD22	BURP domain protein RD22	9.42
RIPt	Beta-galactoside-specific lectin 3	-8.20
Rplp2	60S acidic ribosomal protein P2	10.95
RVE6	Protein REVEILLE 6	1.46

Saa3	Serum amyloid A-3 protein	-9.99
SAG21	Protein SENESCENCE-ASSOCIATED GENE 21, mitochondrial	3.82
SDIR1	E3 ubiquitin-protein ligase SDIR1	-1.75
SIZ1	E3 SUMO-protein ligase SIZ1	4.40
SLE3	Protein SLE3	-1.49
SN1	Snakin-1	3.82
SNAP33	SNAP25 homologous protein SNAP33	3.18
SOT12	Cytosolic sulfotransferase 12	-1.28
SPARC	SPARC	10.29
SRC2	Protein SRC2 homolog	3.98
SRO5	Probable inactive poly [ADP-ribose] polymerase SRO5	2.87
SUS1	Sucrose synthase 1	2.66
TAR2	Tryptophan aminotransferase-related protein 2	1.50
TRX5	Thioredoxin H5	-1.42
TTL4	TPR repeat-containing thioredoxin TTL4	2.88
UGT85K5	Linamarin synthase 2	-3.32
ULP1D	Ubiquitin-like-specific protease 1D	2.71
VQ18	VQ motif-containing protein 18	4.85
VQ20	VQ motif-containing protein 20	1.24
WRKY23	WRKY transcription factor 23	-1.55
WRKY33	Probable WRKY transcription factor 33	3.40
WRKY70	Probable WRKY transcription factor 70	-3.04
WSCP	Kunitz trypsin inhibitor 2	-9.73
**Biotic stress**		
N/A	Non-specific lipid-transfer protein 1	8.82
AAE3	Oxalate--CoA ligase	-12.13
ADF4	Actin-depolymerizing factor 4	-1.01
AGO5	Protein argonaute 5	1.27
AHL20	AT-hook motif nuclear-localized protein 20	-8.55
AMP2-2	Vicilin-like antimicrobial peptides 2-2	-7.21
At3g59930	Defensin-like protein 206	-9.44
At4g20830	Berberine bridge enzyme-like 19	3.19
ATL6	E3 ubiquitin-protein ligase ATL6	-1.44
ATTI1	Defensin-like protein 195	2.86
B2m	Beta-2-microglobulin	-10.25
b2m	Beta-2-microglobulin	9.22
BAG6	BAG family molecular chaperone regulator 6	8.64
BAH1	E3 ubiquitin-protein ligase BAH1	5.27
BBD1	Bifunctional nuclease 1	3.21
BIG5	Brefeldin A-inhibited guanine nucleotide-exchange protein 5	9.13
BIR2	Inactive LRR receptor-like serine/threonine-protein kinase BIR2	8.23
Ca3	Carbonic anhydrase 3	-10.70
CRK8	Cysteine-rich receptor-like protein kinase 8	-1.27
CRN	Inactive leucine-rich repeat receptor-like protein kinase CORYNE	2.89
CYP81D11	Cytochrome P450 81D11	3.93
EDR4	Protein ENHANCED DISEASE RESISTANCE 4	-1.08
eef-2	Elongation factor 2	8.84
ELF5A-2	Eukaryotic translation initiation factor 5A-2	2.14
EXO70B1	Exocyst complex component EXO70B1	9.78
HEL	Hevein-like preproprotein	2.51
HIR3	Hypersensitive-induced response protein 3	2.52

Hp	Haptoglobin	-8.84
KTI1	Kunitz trypsin inhibitor 4	2.42
LYK3	LysM domain receptor-like kinase 3	-1.24
LYK4	LysM domain receptor-like kinase 4	-3.47
Lyz2	Lysozyme C-2	-10.97
MES1	Methylesterase 1	7.05
MLO2	MLO-like protein 2	2.11
NHL10	NDR1/HIN1-like protein 10	3.38
NHL13	NDR1/HIN1-like protein 13	1.84
NHL3	NDR1/HIN1-like protein 3	3.28
OSM34	Osmotin-like protein OSM34	2.91
PBL13	Serine/threonine-protein kinase PBL13	3.21
PCRK1	Serine/threonine-protein kinase PCRK1	-1.58
PDF2.5	Defensin-like protein	4.89
PHOS34	Universal stress protein PHOS34	2.05
PII-2	Piriformospora indica-insensitive protein 2	6.84
PR5K	PR5-like receptor kinase	4.38
RCA	Ribulose bisphosphate carboxylase/oxygenase activase, chloroplastic	3.22
RCA	Ribulose bisphosphate carboxylase/oxygenase activase, chloroplastic	5.41
RLM1A	Disease resistance protein RML1A	3.58
RLP51	Receptor-like protein 51	1.93
SAG20	Senescence associated gene 20	4.23
SDF2	Stromal cell-derived factor 2-like protein	2.99
TET8	Tetraspanin-8	2.23
TIFY10A	Protein TIFY 10A	8.65
TIFY3B	Protein TIFY 3B	2.52
TLP1	Thaumatin-like protein 1	4.38
WIN1	Pathogenesis-related protein PR-4B	-6.33
WRKY17	Probable WRKY transcription factor 17	8.51

Table S8. Translation genes differentially expressed in CR and CS.

Gene name	Protein name	log_2 Ratio (CR/CS)
**Ribosomal protein**		
ARP1	60S ribosomal protein L3-1	-1.77
CTHT_0041970.2	40S ribosomal protein S24	4.27
Os08g0130500	60S acidic ribosomal protein P0	2.10
rpl10	60S ribosomal protein L10	10.85
RPL10A	60S ribosomal protein L10a	-9.37
rpl10a	60S ribosomal protein L10a	12.25
RPL10C	60S ribosomal protein L10-3	2.28
RPL11	60S ribosomal protein L11	-9.30
rpl11	60S ribosomal protein L11	12.07
RPL12	60S ribosomal protein L12	12.13
rpl1202	60S ribosomal protein L12-B	7.78
RPL12A	50S ribosomal protein L12-1, chloroplastic	8.87
RPL13	60S ribosomal protein L13	8.23
Rpl13	60S ribosomal protein L13	12.15
RPL13A	60S ribosomal protein L13a	11.82
RPL14	60S ribosomal protein L14	10.88
RPL15	60S ribosomal protein L15	-8.47
rpl15	60S ribosomal protein L15	11.74
rpl16	50S ribosomal protein L16, plastid	-1.03
RPL17	60S ribosomal protein L17	-9.43
Rpl17	60S ribosomal protein L17	12.57
RPL18	50S ribosomal protein L18, chloroplastic	1.11
rpl18	60S ribosomal protein L18	11.55
rpl18a	60S ribosomal protein L18a	11.83
RPL19	60S ribosomal protein L19	-8.57
rpl19	60S ribosomal protein L19	10.89
RPL21	60S ribosomal protein L21	11.06
Rpl22	60S ribosomal protein L22	10.70
RPL22B	60S ribosomal protein L22-2	2.14
RPL23	60S ribosomal protein L23	12.86
RPL23A	60S ribosomal protein L23A	2.95
RPL23A	60S ribosomal protein L23a	12.63
RPL24	60S ribosomal protein L24	11.61
RPL24B	60S ribosomal protein L24-2	2.08
RPL26	60S ribosomal protein L26	5.02
RPL26	60S ribosomal protein L26	12.27
rpl27	60S ribosomal protein L27	11.08
RPL27A	60S ribosomal protein L27a	4.74
RPL27A	60S ribosomal protein L27a	11.64
RPL27A	60S ribosomal protein L27a	-9.23
RPL28	50S ribosomal protein L28, chloroplastic	1.60
Rpl28	60S ribosomal protein L28	12.10
RPL29	60S ribosomal protein L29	10.86
RPL3	60S ribosomal protein L3	11.86
RPL3	Ribosomal protein L3	1.85
rpl30	60S ribosomal protein L30	11.83

RPL30	60S ribosomal protein L30	8.92
RPL31	60S ribosomal protein L31	-9.24
rpl31	60S ribosomal protein L31	12.10
rpl32	60S ribosomal protein L32	11.86
rpl34	60S ribosomal protein L34	11.05
rpl35	60S ribosomal protein L35	12.35
RPL35	60S ribosomal protein L35	-2.02
rpl35a	60S ribosomal protein L35a	11.09
rpl36	60S ribosomal protein L36	11.24
rpl36a	60S ribosomal protein L36a	7.12
rpl37	60S ribosomal protein L37	11.66
Rpl37	60S ribosomal protein L37	-9.00
RPL37A	60S ribosomal protein L37a	-8.70
rpl37a	60S ribosomal protein L37a	11.95
RPL38	60S ribosomal protein L38	2.80
rpl39	60S ribosomal protein L39	10.92
RPL4	60S ribosomal protein L4	2.93
RPL4	60S ribosomal protein L4	-7.65
rpl4-a	60S ribosomal protein L4-A	11.30
RPL5	60S ribosomal protein L5	3.51
RPL5	60S ribosomal protein L5	10.98
RPL5	60S ribosomal protein L5, mitochondrial	2.97
RPL6	60S ribosomal protein L6	2.75
RPL6	60S ribosomal protein L6	11.93
RPL6C	60S ribosomal protein L6-3	3.22
RPL7	60S ribosomal protein L7	11.53
rpl7a	60S ribosomal protein L7a	12.32
RPL7A	60S ribosomal protein L7a	-7.77
RPL8	60S ribosomal protein L8	3.10
rpl8	60S ribosomal protein L8	11.52
RPL8	60S ribosomal protein L8	-9.31
rpl9	60S ribosomal protein L9	11.54
RPLP0	60S acidic ribosomal protein P0	-7.39
rplp0	60S acidic ribosomal protein P0	11.88
RPLP1	60S acidic ribosomal protein P1	12.80
RPLP1	60S acidic ribosomal protein P1	3.12
Rplp1	60S acidic ribosomal protein P1	-9.36
RPLP1	60S acidic ribosomal protein P1	-9.72
RPLP2	60S acidic ribosomal protein P2	11.30
RPP1A	60S acidic ribosomal protein P1	2.65
RPP1A	60S acidic ribosomal protein P1	1.03
Rps10	40S ribosomal protein S10	-8.04
RPS10	40S ribosomal protein S10	-8.88
rps10	40S ribosomal protein S10	11.47
RPS10A	40S ribosomal protein S10-1	3.46
rps11	30S ribosomal protein S11, chloroplastic	-6.25
RPS11	40S ribosomal protein S11	11.66
RPS12	40S ribosomal protein S12	11.38
RPS12	40S ribosomal protein S12	4.54
RPS12	40S ribosomal protein S12	-8.36
rps13	40S ribosomal protein S13	11.69

RPS14	40S ribosomal protein S14	7.35
RPS14A	40S ribosomal protein S14-1	3.08
RPS14B	40S ribosomal protein S14-2	2.65
RPS15	40S ribosomal protein S15	10.89
RPS15A	40S ribosomal protein S15a	11.23
RPS16	40S ribosomal protein S16	-9.00
rps16	40S ribosomal protein S16	11.48
RPS17	40S ribosomal protein S17	9.82
rps18	40S ribosomal protein S18	12.15
RPS18B	40S ribosomal protein S18-B	3.02
rps19	40S ribosomal protein S19	12.92
RPS2	40S ribosomal protein S2	-9.07
RpS2	40S ribosomal protein S2	2.97
rps2	40S ribosomal protein S2	12.46
RPS20	40S ribosomal protein S20	12.88
rps21	40S ribosomal protein S21	9.86
RPS21C	40S ribosomal protein S21-2	1.07
RPS23	40S ribosomal protein S12	10.07
rps23	40S ribosomal protein S23	11.57
RPS24	40S ribosomal protein S24	-8.69
rps24	40S ribosomal protein S24	12.12
rps25	40S ribosomal protein S25	11.03
RPS26	40S ribosomal protein S26	11.48
rps27	40S ribosomal protein S27	11.24
Rps27	40S ribosomal protein S27	10.79
rps27a	Ubiquitin-40S ribosomal protein S27a	11.18
RPS27L	40S ribosomal protein S27-like	10.35
rps28	40S ribosomal protein S28	10.07
rps3	40S ribosomal protein S3	11.27
RPS3A	40S ribosomal protein S3a	-9.18
rps3a	40S ribosomal protein S3a	12.21
RPS4X	40S ribosomal protein S4, X isoform	-7.50
rps4x	40S ribosomal protein S4, X isoform	9.01
RPS5	40S ribosomal protein S5	11.04
RPS6	40S ribosomal protein S6	-9.33
rps6	40S ribosomal protein S6	12.56
RPS6	40S ribosomal protein S6	-3.48
rps7	40S ribosomal protein S7	11.33
RPS7	40S ribosomal protein S7	-9.51
RPS7	Ribosomal protein S7, mitochondrial	1.04
rps8	40S ribosomal protein S8	11.76
RPS8	40S ribosomal protein S8	-9.35
RPS9	40S ribosomal protein S9	11.58
RPSA	40S ribosomal protein SA	-8.98
RPSA	40S ribosomal protein SA	-9.22
rpsa	40S ribosomal protein SA	11.66
rpsO	30S ribosomal protein S15	-1.48
Tpt1	Translationally-controlled tumor protein	-10.13
TPT1	Translationally-controlled tumor protein	-9.91
UBA52	Ubiquitin-60S ribosomal protein L40	7.47
ubi3	Ubiquitin-40S ribosomal protein S27a	2.30

YRPS3	Ribosomal protein S3, mitochondrial	-1.67
**Translation initiation factor**		
At1g54290	Protein translation factor SUI1 homolog 2	3.17
At1g77840	Probable eukaryotic translation initiation factor 5-2	4.36
At4g27130	Protein translation factor SUI1 homolog 1	4.62
EIF(ISO)4G1	Eukaryotic translation initiation factor isoform 4G-1	2.95
Eif1	Eukaryotic translation initiation factor 1	-8.88
EIF4B3	Eukaryotic translation initiation factor 4B3	-1.26
EIF5A	Eukaryotic translation initiation factor 5A-1	10.64
EIF5A	Eukaryotic translation initiation factor 5A-1	-8.73
EIF-5A1	Eukaryotic translation initiation factor 5A	3.17
ERF1-1	Eukaryotic peptide chain release factor subunit 1-1	4.80
TIF	Protein translation factor SUI1 homolog	4.96
TIF11	Eukaryotic translation initiation factor 1A	2.38
TIF3C1	Eukaryotic translation initiation factor 3 subunit C	9.03
TIF4A-1	Eukaryotic initiation factor 4A-1	2.35
**Elongation factor**		
At1g30230	Elongation factor 1-delta	-1.65
At1g30230	Elongation factor 1-delta 1	2.43
eef1a	Elongation factor 1-alpha	13.65
EEF1A1	Elongation factor 1-alpha 1	-8.10
EEF1B	Elongation factor 1-beta	11.10
EEF1G	Elongation factor 1-gamma	-9.16
eef1g	Elongation factor 1-gamma	11.42
Eef2	Elongation factor 2	-8.97
EEF2	Elongation factor 2	11.72
EEF2	Elongation factor 2	11.65
Ef1alpha100E	Elongation factor 1-alpha	5.97
LOS1	Elongation factor 2	1.93
TEF	Elongation factor 1-alpha	2.28
tef2	Translation elongation factor 2	8.60
others		
APUM11	Pumilio homolog 11	2.56
APUM12	Pumilio homolog 12	4.96
At3g62120	Proline--tRNA ligase, cytoplasmic	2.30
At5g08100	Isoaspartyl peptidase/L-asparaginase 1	1.02
At5g26710	Glutamate--tRNA ligase, cytoplasmic	3.08
CAF1-7	Probable CCR4-associated factor 1 homolog 7	2.65
CNR7	Cell number regulator 7	-1.54
FAU	Ubiquitin-like protein FUBI	11.42
fes1	Hsp70 nucleotide exchange factor fes1	4.63
LSG1-2	GTPase LSG1-2	1.80
N/A	Protein phosphatase PP2A regulatory subunit A	3.30
NARS	Asparagine--tRNA ligase, cytoplasmic	10.33
PAB7	Polyadenylate-binding protein 7	2.84
PEL1	Protein PELOTA 1	-6.50
ran	GTP-binding nuclear protein Ran	9.81
RNP1	Heterogeneous nuclear ribonucleoprotein 1	1.89
rsl24d1	Probable ribosome biogenesis protein RLP24	9.09
SYNC1	Asparagine--tRNA ligase, cytoplasmic 1	3.18

Table S9. Secondary metabolism genes differentially expressed in CR and CS.

Gene name	Protein name	log_2 Ratio (CR/CS)
**Hormone biosynthesis and metabolism**		
N/A	Zeaxanthin epoxidase, chloroplastic	1.06
ABA2	Xanthoxin dehydrogenase	1.61
ACO	1-aminocyclopropane-1-carboxylate oxidase	-1.16
ACO1	1-aminocyclopropane-1-carboxylate oxidase	-4.59
ACO3	1-aminocyclopropane-1-carboxylate oxidase 3	-4.01
ACS1	1-aminocyclopropane-1-carboxylate synthase	-2.52
ACS-1	1-aminocyclopropane-1-carboxylate synthase	10.14
ACS8	1-aminocyclopropane-1-carboxylate synthase 8	1.70
CKX1	Cytokinin dehydrogenase 1	4.03
CKX5	Cytokinin dehydrogenase 5	3.19
CKX6	Cytokinin dehydrogenase 6	4.83
CKX7	Cytokinin dehydrogenase 7	-1.46
CYP707A1	Abscisic acid 8'-hydroxylase 1	-2.24
CYP707A4	Abscisic acid 8'-hydroxylase 4	1.16
CYP707A6	Abscisic acid 8'-hydroxylase 2	-1.04
CYP735A2	Cytokinin hydroxylase	-3.20
GA1	Ent-copalyl diphosphate synthase, chloroplastic	2.08
GA20OX1	Gibberellin 20 oxidase 1	6.34
GA2OX1	Gibberellin 2-beta-dioxygenase	1.34
GA2OX2	Gibberellin 2-beta-dioxygenase 2	5.62
GA2OX6	Gibberellin 2-beta-dioxygenase 6	2.72
GULLO3	L-gulonolactone oxidase 3	1.04
GULLO6	Probable L-gulonolactone oxidase 6	2.38
HSD3B	3 beta-hydroxysteroid dehydrogenase/Delta 5-->4-isomerase	-8.95
IPT5	Adenylate isopentenyltransferase 5, chloroplastic	-11.35
LE	Gibberellin 3-beta-dioxygenase 1	-1.36
LOG3	Cytokinin riboside 5'-monophosphate phosphoribohydrolase LOG3	2.44
LOG7	Cytokinin riboside 5'-monophosphate phosphoribohydrolase LOG7	10.47
LOG8	Cytokinin riboside 5'-monophosphate phosphoribohydrolase LOG8	-3.45
NPF3.1	Protein NRT1/ PTR FAMILY 3.1	-4.18
WAT1	Protein WALLS ARE THIN 1	1.77
XERICO	Probable E3 ubiquitin-protein ligase XERICO	1.83
YUC10	Probable indole-3-pyruvate monooxygenase YUCCA10	-5.41
YUC4	Probable indole-3-pyruvate monooxygenase YUCCA4	9.41
YUC6	Indole-3-pyruvate monooxygenase YUCCA6	10.20
**Terpene biosynthesis and metabolism**		
CYC2	Iridoid synthase	-2.09
CYC2	Iridoid synthase CYC2	-1.08
CYP716A17	Beta-amyrin 28-monooxygenase	2.04
GGR	Heterodimeric geranylgeranyl pyrophosphate synthase small subunit, chloroplastic	2.37
LUS1	Lupeol synthase	-4.48
OSCBPW	Lupeol synthase	-3.66
VIT_19s0014g0493	(-)-germacrene D synthase	3.66

0		
**Flavonoid biosynthesis and metabolism**		
ANR	Anthocyanidin reductase ((2S)-flavan-3-ol-forming)	-1.76
At3g11180	Probable 2-oxoglutarate-dependent dioxygenase At3g111800	-4.87
BHLH	Basic helix-loop-helix protein A	-1.32
CHI	Chalcone--flavonone isomerase	-1.35
DTX35	DETOXIFICATION 35	1.61
DTX41	Protein DETOXIFICATION 41	-1.97
FHT	Naringenin,2-oxoglutarate 3-dioxygenase	-1.95
HIDM	2-hydroxyisoflavanone dehydratase	-2.72
**Jasmonic acid biosynthesis**		
4CLL5	4-coumarate--CoA ligase-like 5	4.42
AOS1	Allene oxide synthase 1, chloroplastic	-3.29
GRXC9	Glutaredoxin-C9	-2.72
MES17	Methylesterase 17	2.64
OPR3	12-oxophytodienoate reductase 3	-1.41
**Alkaloid metabolism**		
AAE	Acetylajmalan esterase	-1.59
ACT	Vinorine synthase	-2.97
CTS2	Theobromine synthase 2	3.47
TCS2	Probable caffeine synthase 2	-1.78
**Hydrogen peroxide catabolism**		
PER10	Peroxidase 10	1.34
PER12	Peroxidase 12	2.17
PER17	Peroxidase 17	1.88
PER43	Peroxidase 43	-1.74
PER47	Peroxidase 47	3.22
PER54	Peroxidase 54	4.55
PER64	Peroxidase 64	1.07
PER69	Peroxidase 69	-4.80
PNC1	Cationic peroxidase 1	-1.42
PNC2	Cationic peroxidase 2	-1.30
PRXC2	Peroxidase C2	6.86
**Others**		
10HGO	8-hydroxygeraniol dehydrogenase	1.46
4CL1	4-coumarate--CoA ligase 1	-1.04
4CLL6	4-coumarate--CoA ligase-like 6	5.14
ADC2	Arginine decarboxylase 2	9.02
adhC2	NADP-dependent alcohol dehydrogenase C 2	2.02
AHA10	ATPase 10, plasma membrane-type	-1.83
AKR1C1	Prostaglandin F synthase 1	-10.20
ALDH	Aldehyde dehydrogenase	-3.05
ALDH2C4	Aldehyde dehydrogenase family 2 member C4	-5.19
ALN	Allantoinase	1.33
ANS	Leucoanthocyanidin dioxygenase	-1.14
AOX4	Ubiquinol oxidase 4, chloroplastic/chromoplastic	1.32
At1g10310	NADPH-dependent pterin aldehyde reductase	1.50
At1g73050	(R)-mandelonitrile lyase-like	1.11
At2g04865	MAIN-LIKE 2	-9.21
At5g24760	Alcohol dehydrogenase-like 6	-1.29
At5g38780	Probable S-adenosylmethionine-dependent	-4.62

	methyltransferase At5g38780	
At5g60760	P-loop NTPase domain-containing protein LPA1 homolog 1	-3.68
ATR2	NADPH--cytochrome P450 reductase 2	2.35
BCA1	Beta carbonic anhydrase 1, chloroplastic	5.05
BCA2	Beta carbonic anhydrase 2, chloroplastic	9.57
BCA2	Carbonic anhydrase, chloroplastic	-1.27
BEAT	Acetyl-CoA-benzylalcohol acetyltransferase	-10.99
Bp10	L-ascorbate oxidase homolog	-2.72
CAD9	Probable cinnamyl alcohol dehydrogenase 9	-3.03
CAT1	Catalase isozyme 1	-3.62
CER3	Very-long-chain aldehyde decarbonylase CER3	1.72
CRY1	Cryptochrome-1	1.95
CSE	Caffeoylshikimate esterase	-3.56
CXE15	Probable carboxylesterase 15	1.80
CYN	Cyanate hydratase	1.26
CYN	Cyanate hydratase	3.23
CYP716A15	Beta-amyrin 28-monooxygenase	-3.59
CYP71D8	Germacrene A hydroxylase	-1.31
CYP79A1	Tyrosine N-monooxygenase	-3.98
CYP79D4	Isoleucine N-monooxygenase 2	-4.06
CYP96A15	Alkane hydroxylase MAH1	-3.82
Dctpp1	dCTP pyrophosphatase 1	-1.63
DFR	Dihydroflavonol 4-reductase	-1.58
DFRA	Dihydroflavonol 4-reductase	-2.18
DGAT1	Diacylglycerol O-acyltransferase 1	-1.26
DGAT3	Diacylglycerol O-acyltransferase 3	3.99
DJ1B	Protein DJ-1 homolog B	4.18
DLO2	Protein DMR6-LIKE OXYGENASE 2	-1.43
DSEL	Phospholipase A1-IIgamma	2.75
ERF9	Ethylene-responsive transcription factor 9	2.30
F6'H1	Feruloyl CoA ortho-hydroxylase 1	-4.24
FDH1	Formate dehydrogenase, chloroplastic/mitochondrial	8.86
FHY	Bifunctional riboflavin kinase/FMN phosphatase	1.05
FLS	Flavonol synthase/flavanone 3-hydroxylase	8.60
FMOGS-OX3	Flavin-containing monooxygenase FMO GS-OX3	-3.06
GDPD1	Glycerophosphodiester phosphodiesterase GDPD1, chloroplastic	-2.84
GGP1	Gamma-glutamyl peptidase 1	4.15
GGPS	Geranylgeranyl pyrophosphate synthase, chloroplastic	1.54
GPAT1	Glycerol-3-phosphate acyltransferase 1	5.99
GPX3	Glutathione peroxidase 3	-11.36
GRF1	Growth-regulating factor 1	3.43
GT5	Anthocyanidin 3-O-glucosyltransferase 5	-6.85
HIPL1	HIPL1 protein	1.69
HMG1	3-hydroxy-3-methylglutaryl-coenzyme A reductase 1	2.99
HMGS	Hydroxymethylglutaryl-CoA synthase	-1.69
HRPN	Peroxidase N	-1.73
ICDH-1	Isocitrate dehydrogenase [NADP]	-1.33
IGMT4	Indole glucosinolate O-methyltransferase 4	2.61
IMPDH	Inosine-5'-monophosphate dehydrogenase 1	4.07
Inmt	Indolethylamine N-methyltransferase	-8.65

IP5P4	Type I inositol polyphosphate 5-phosphatase 4	1.26
IPCS2	Phosphatidylinositol:ceramide inositolphosphotransferase 2	-1.41
ISPH	4-hydroxy-3-methylbut-2-enyl diphosphate reductase, chloroplastic	3.24
KES1	Protein KES1	3.41
LOX3.1	Linoleate 13S-lipoxygenase 3-1, chloroplastic	-3.84
malA	NADP-dependent malic enzyme	2.10
MED33A	Mediator of RNA polymerase II transcription subunit 33A	-1.67
MEE23	Berberine bridge enzyme-like 15	2.13
NADK3	NADH kinase	3.67
NDPK2	Nucleoside diphosphate kinase 2, chloroplastic	-1.25
NIA1	Nitrate reductase [NADH], clone PBNBR1405	3.85
NIR1	Ferredoxin--nitrite reductase, chloroplastic	4.42
NIT4	Bifunctional nitrilase/nitrile hydratase NIT4	8.31
NLP2	Deaminated glutathione amidase, chloroplastic/cytosolic	-1.13
NME1-2	Nucleoside diphosphate kinase A2	11.55
NMRK2	Nicotinamide riboside kinase 2	11.54
NSP5	Nitrile-specifier protein 5	4.91
NTR2	Thioredoxin reductase 2	2.89
OMT1	Flavone 3'-O-methyltransferase 1	-1.73
Os01g0789100	CDP-diacylglycerol--glycerol-3-phosphate 3-phosphatidyltransferase 1, chloroplastic	1.21
Os04g0670700	Probable phytol kinase, chloroplastic	1.01
OVA9	Glutamine--tRNA ligase, cytoplasmic	3.90
PAO3	Polyamine oxidase 3	-1.68
PCBER	Phenylcoumaran benzylic ether reductase POP1	1.38
PDC4	Pyruvate decarboxylase 4	3.41
PSY	Phytoene synthase, chloroplastic	2.50
ROMT	Trans-resveratrol di-O-methyltransferase	-4.58
RUB1	Ubiquitin-NEDD8-like protein RUB1	3.87
SCPL18	Serine carboxypeptidase-like 18	-1.12
SDC	Serine decarboxylase	1.04
Sftpb	Pulmonary surfactant-associated protein B	-8.72
SMT2	24-methylenesterol C-methyltransferase 2	1.17
SNL6	Cinnamoyl-CoA reductase-like SNL6	-1.96
STPS	Sesquiterpene synthase	9.19
SUD1	Probable E3 ubiquitin ligase SUD1	5.09
TBL2	Protein trichome birefringence-like 2	1.61
TBL38	Protein trichome birefringence-like 38	-2.66
TBL8	Protein trichome birefringence-like 8	1.60
THI1	Thiamine thiazole synthase, chloroplastic	3.70
THIC	Phosphomethylpyrimidine synthase, chloroplastic	4.25
TP53I3	Quinone oxidoreductase PIG3	2.69
TPI1	Triosephosphate isomerase	-6.83
TSPO	Translocator protein homolog	-1.18

Table S10. Photosynthesis and energy genes differentially expressed in CR and CS.

Gene name	Protein name	log_2 Ratio (CR/CS)
**Photosynthesis and energy**		
AOX1A	Ubiquinol oxidase 1a, mitochondrial	3.00
BCA4	Beta carbonic anhydrase 4	3.31
CHLH	Magnesium-chelatase subunit ChlH, chloroplastic	2.16
CHLI	Magnesium-chelatase subunit ChlI, chloroplastic	1.33
CHLM	Magnesium protoporphyrin IX methyltransferase, chloroplastic	2.00
CHLP	Geranylgeranyl diphosphate reductase, chloroplastic	4.38
CHLP	Geranylgeranyl diphosphate reductase, chloroplastic	2.14
CPX1	Coproporphyrinogen-III oxidase 1, chloroplastic	4.09
CRD1	Magnesium-protoporphyrin IX monomethyl ester [oxidative] cyclase, chloroplastic	1.34
CRD1	Magnesium-protoporphyrin IX monomethyl ester [oxidative] cyclase, chloroplastic	4.38
DCUP	Uroporphyrinogen decarboxylase, chloroplastic	-1.86
DHNAT1	1,4-dihydroxy-2-naphthoyl-CoA thioesterase 1	-1.12
DVR	Divinyl chlorophyllide a 8-vinyl-reductase, chloroplastic	2.64
EFL4	Protein ELF4-LIKE 4	1.88
FAR1	Protein FAR-RED IMPAIRED RESPONSE 1	-2.31
FD2	Ferredoxin-2, chloroplastic	3.33
FTSH1	ATP-dependent zinc metalloprotease FTSH 1, chloroplastic	1.29
FTSH1	ATP-dependent zinc metalloprotease FTSH 1, chloroplastic	1.22
GLO1	(S)-2-hydroxy-acid oxidase GLO1	4.94
GLYK	D-glycerate 3-kinase, chloroplastic	3.13
HY5	Transcription factor HY5	-1.67
KAI2	Probable esterase KAI2	3.39
MTACP2	Acyl carrier protein 3, mitochondrial	-1.85
mt-atp6	ATP synthase subunit a	12.81
MT-ND1	NADH-ubiquinone oxidoreductase chain 1	-5.02
MT-ND1	NADH-ubiquinone oxidoreductase chain 1	12.05
MT-ND2	NADH-ubiquinone oxidoreductase chain 2	-8.73
MT-ND3	NADH-ubiquinone oxidoreductase chain 3	10.74
MT-ND4	NADH-ubiquinone oxidoreductase chain 4	11.14
MT-ND4	NADH-ubiquinone oxidoreductase chain 4	-10.16
MT-ND5	NADH-ubiquinone oxidoreductase chain 5	10.28
MT-ND6	NADH-ubiquinone oxidoreductase chain 6	-9.67
ND2	NADH-ubiquinone oxidoreductase chain 2	-1.62
PAT1	Scarecrow-like transcription factor PAT1	1.12
PDR2	Pleiotropic drug resistance protein 2	-1.35
PFP-ALPHA2	Pyrophosphate--fructose 6-phosphate 1-phosphotransferase subunit alpha 2	2.40
PORA	Protochlorophyllide reductase, chloroplastic	1.58
PSBO1	Oxygen-evolving enhancer protein 1-1, chloroplastic	8.39
PSBP1	Oxygen-evolving enhancer protein 2-1, chloroplastic	4.92
RBCS-1A	Ribulose bisphosphate carboxylase small chain 1A, chloroplastic	5.37
RBCS-2	Ribulose bisphosphate carboxylase small chain 2, chloroplastic	10.30
RBCS-3B	Ribulose bisphosphate carboxylase small chain 3B, chloroplastic	9.90
SPA3	Protein SPA1-RELATED 3	1.04
UVR8	Ultraviolet-B receptor UVR8	1.17

ychF	Ribosome-binding ATPase YchF	1.00
**Photosystem I and II**		
LHCA2	Photosystem I chlorophyll a/b-binding protein 2, chloroplastic	9.33
LHCA3	Photosystem I chlorophyll a/b-binding protein 3-1, chloroplastic	10.01
psaD1	Photosystem I reaction center subunit II-1, chloroplastic	9.74
PSAE1	Photosystem I reaction center subunit IV A, chloroplastic	5.56
PSAF	Photosystem I reaction center subunit III, chloroplastic	4.88
PSAG	Photosystem I reaction center subunit V, chloroplastic	10.16
PSAK	Photosystem I reaction center subunit psaK, chloroplastic	5.53
PSAL	Photosystem I reaction center subunit XI, chloroplastic	4.73
PSAN	Photosystem I reaction center subunit N, chloroplastic	4.46
PSAO	Photosystem I subunit O	9.62
PSBR	Photosystem II 10 kDa polypeptide, chloroplastic	4.13
PSBS	Photosystem II 22 kDa protein, chloroplastic	10.22
PSBT	Photosystem II 5 kDa protein, chloroplastic	10.21
PSBW	Photosystem II reaction center W protein, chloroplastic	4.21
PSBY	Photosystem II core complex proteins psbY, chloroplastic	10.20
**Chlorophyll binding protein**		
CAB1	Chlorophyll a-b binding protein 1, chloroplastic	5.69
cabII-1	Chlorophyll a-b binding protein of LHCII type I, chloroplastic	11.49
CAP10B	Chlorophyll a-b binding protein CP24 10B, chloroplastic	9.41
LHCA1	Chlorophyll a-b binding protein 6, chloroplastic	5.32
LHCA4	Chlorophyll a-b binding protein 4, chloroplastic	5.48
LHCB1.3	Chlorophyll a-b binding protein 1, chloroplastic	6.60
LHCB2.1	Chlorophyll a-b binding protein 2.1, chloroplastic	11.23
LHCB2.1	Chlorophyll a-b binding protein, chloroplastic	8.56
LHCB2.4	Chlorophyll a-b binding protein 2.4, chloroplastic	7.55
LHCB3	Chlorophyll a-b binding protein 3, chloroplastic	10.09
LHCB4.2	Chlorophyll a-b binding protein CP29.2, chloroplastic	9.76
LHCB5	Chlorophyll a-b binding protein CP26, chloroplastic	5.21
OsI_012078	Chlorophyll a-b binding protein, chloroplastic	7.03
**Cytochrome**		
CB5-A	Cytochrome b5 isoform A	1.25
CCMFC	Cytochrome c biogenesis CcmF C-terminal-like mitochondrial protein	-1.75
CCMH	Cytochrome c-type biogenesis CcmH-like mitochondrial protein	-1.08
COX1	Cytochrome c oxidase subunit 1	-2.47
COX15	Cytochrome c oxidase assembly protein COX15	1.12
CYB5	Cytochrome b5	3.42
Cyp2f2	Cytochrome P450 2F2	-7.82
CYP704C1	Cytochrome P450 704C1	-1.05
CYP705A12	Cytochrome P450 705A12	1.14
CYP714C2	Cytochrome P450 714C2	5.94
CYP71A9	Cytochrome P450 71A9	-3.44
CYP71D10	Cytochrome P450 71D10	-2.75
CYP71D9	Cytochrome P450 71D9	-3.70
CYP734A1	Cytochrome P450 734A1	2.61
CYP750A1	Cytochrome P450 CYP736A12	-3.06
CYP77A2	Cytochrome P450 77A2	1.90
CYP77A3	Cytochrome P450 77A3	11.26
CYP78A7	Cytochrome P450 78A7	4.71

CYP78A9	Cytochrome P450 78A9	-4.81
CYP90A1	Cytochrome P450 90A1	1.20
CYP90B1	Cytochrome P450 90B1	3.64
CYP93A3	Cytochrome P450 93A3	2.77
CYP94B1	Cytochrome P450 94B1	-6.81
CYP98A2	Cytochrome P450 98A2	-1.71
Mtco1	Cytochrome c oxidase subunit 1	-12.59
mt-co1	Cytochrome c oxidase subunit 1	13.37
MT-CO1	Cytochrome c oxidase subunit 1	-13.24
mt-co2	Cytochrome c oxidase subunit 2	13.36
MT-CO2	Cytochrome c oxidase subunit 2	-7.01
MT-CO2	Cytochrome c oxidase subunit 2	-11.88
mt-co3	Cytochrome c oxidase subunit 3	13.44
mt-Co3	Cytochrome c oxidase subunit 3	-12.45
MT-CO3	Cytochrome c oxidase subunit 3	-11.80
mt-cyb	Cytochrome b	12.48
Mt-Cyb	Cytochrome b	-11.28
petC	Cytochrome b6-f complex iron-sulfur subunit, chloroplastic	9.01
